# Bacterial defenses against a natural antibiotic promote collateral resilience to clinical antibiotics

**DOI:** 10.1371/journal.pbio.3001093

**Published:** 2021-03-10

**Authors:** Lucas A. Meirelles, Elena K. Perry, Megan Bergkessel, Dianne K. Newman

**Affiliations:** 1 Division of Biology and Biological Engineering, California Institute of Technology, Pasadena, California, United States of America; 2 Division of Geological and Planetary Sciences, California Institute of Technology, Pasadena, California, United States of America; Hebrew University, ISRAEL

## Abstract

Bacterial opportunistic human pathogens frequently exhibit intrinsic antibiotic tolerance and resistance, resulting in infections that can be nearly impossible to eradicate. We asked whether this recalcitrance could be driven by these organisms’ evolutionary history as environmental microbes that engage in chemical warfare. Using *Pseudomonas aeruginosa* as a model, we demonstrate that the self-produced antibiotic pyocyanin (PYO) activates defenses that confer collateral tolerance specifically to structurally similar synthetic clinical antibiotics. Non-PYO-producing opportunistic pathogens, such as members of the *Burkholderia cepacia* complex, likewise display elevated antibiotic tolerance when cocultured with PYO-producing strains. Furthermore, by widening the population bottleneck that occurs during antibiotic selection and promoting the establishment of a more diverse range of mutant lineages, PYO increases apparent rates of mutation to antibiotic resistance to a degree that can rival clinically relevant hypermutator strains. Together, these results reveal an overlooked mechanism by which opportunistic pathogens that produce natural toxins can dramatically modulate the efficacy of clinical antibiotics and the evolution of antibiotic resistance, both for themselves and other members of clinically relevant polymicrobial communities.

## Abstract 2

This study shows that pyocyanin, a toxin secreted by the opportunistic pathogen Pseudomonas aeruginosa, induces defense responses that decrease the efficacy of structurally-similar clinical antibiotics and accelerate the evolution of antibiotic resistance, both in the producer and in other members of clinically-relevant polymicrobial communities.

## Introduction

The emergence and spread of bacterial resistance to clinical antibiotics is a growing public health concern worldwide [1]. Moreover, it is increasingly appreciated that antibiotic tolerance can also contribute to the failure of treatments for infections [2] and that tolerance can lead to the evolution of resistance [3,4]. Yet bacterial resilience to antibiotics is anything but new: Microbes in environments like soil have been producing natural antibiotics and evolving mechanisms of tolerance and resistance for millions of years [5,6]. Here, we define tolerance as the ability to survive a transient exposure to an otherwise lethal antibiotic concentration and resistance as the ability to grow in the presence of an antibiotic, similar to recent recommendations [2,7,8].

Considering that most of the antibiotics used today are derived from microbially produced molecules, we hypothesized that molecular defenses that originally evolved to protect cells from a natural antibiotic in the environment might also promote tolerance and/or resistance to structurally or mechanistically similar clinical drugs. Indeed, several clinical antibiotic resistance genes are thought to have originated in nonpathogenic soil bacteria, but it has often been assumed that intermediate steps of horizontal gene transfer are necessary in order for such genes to be acquired by human pathogens [6]. In this study, we asked whether there could be a direct link between production of natural antibiotics by an opportunistic human pathogen and its recalcitrance to clinical antibiotic treatment due to shared protective mechanisms. In addition, we sought to determine whether in the presence of such a natural antibiotic producer, recalcitrance to clinical antibiotics could also be observed in other opportunistic pathogens found together with it in polymicrobial infections. Given that many opportunistic pathogens share their natural environment (e.g., soil), we posited that the evolutionary legacy of natural antibiotic–mediated ecological interactions between these microbial species could have important implications for antibiotic tolerance and resistance in the clinical context.

One organism that is well suited to testing these hypotheses is the opportunistic pathogen *Pseudomonas aeruginosa*, which is notorious for causing chronic lung infections in cystic fibrosis (CF) patients, as well as other types of infections in immunocompromised hosts [9]. *P*. *aeruginosa* produces several redox-active, heterocyclic compounds known as phenazines [10]. Phenazines have been shown to provide multiple benefits for their producers, including by (i) serving as an alternative electron acceptor in the absence of oxygen, thereby promoting redox homeostasis and anaerobic survival [11], which is particularly relevant for oxidant-limited biofilms [12]; (ii) acting as signaling molecules [13]; (iii) promoting iron acquisition [14]; and (iv) killing competitor species [15]. In addition, despite possessing broad-spectrum antimicrobial activity [10], including against *P*. *aeruginosa* itself [16], phenazines have recently been shown to promote tolerance to clinical antibiotics under some circumstances, via mechanisms that have yet to be characterized [17,18]. Here, we sought to assess potential broader implications of this phenomenon by investigating whether phenazine-mediated tolerance to clinical antibiotics in *P*. *aeruginosa* is driven by cellular defenses that evolved to mitigate self-induced toxicity. We also tested whether phenazine production by *P*. *aeruginosa* could promote antibiotic tolerance in other clinically relevant opportunistic pathogens from the *Burkholderia* and *Stenotrophomonas* genera. Finally, we explored the ramifications of phenazine-induced tolerance for the evolution of heritable antibiotic resistance, both in *P*. *aeruginosa* and in a clinical isolate from the *Burkholderia cepacia* complex.

## Results

### Mechanisms of tolerance to the self-produced natural antibiotic PYO in *P*. *aeruginosa*

**Fig 1:**
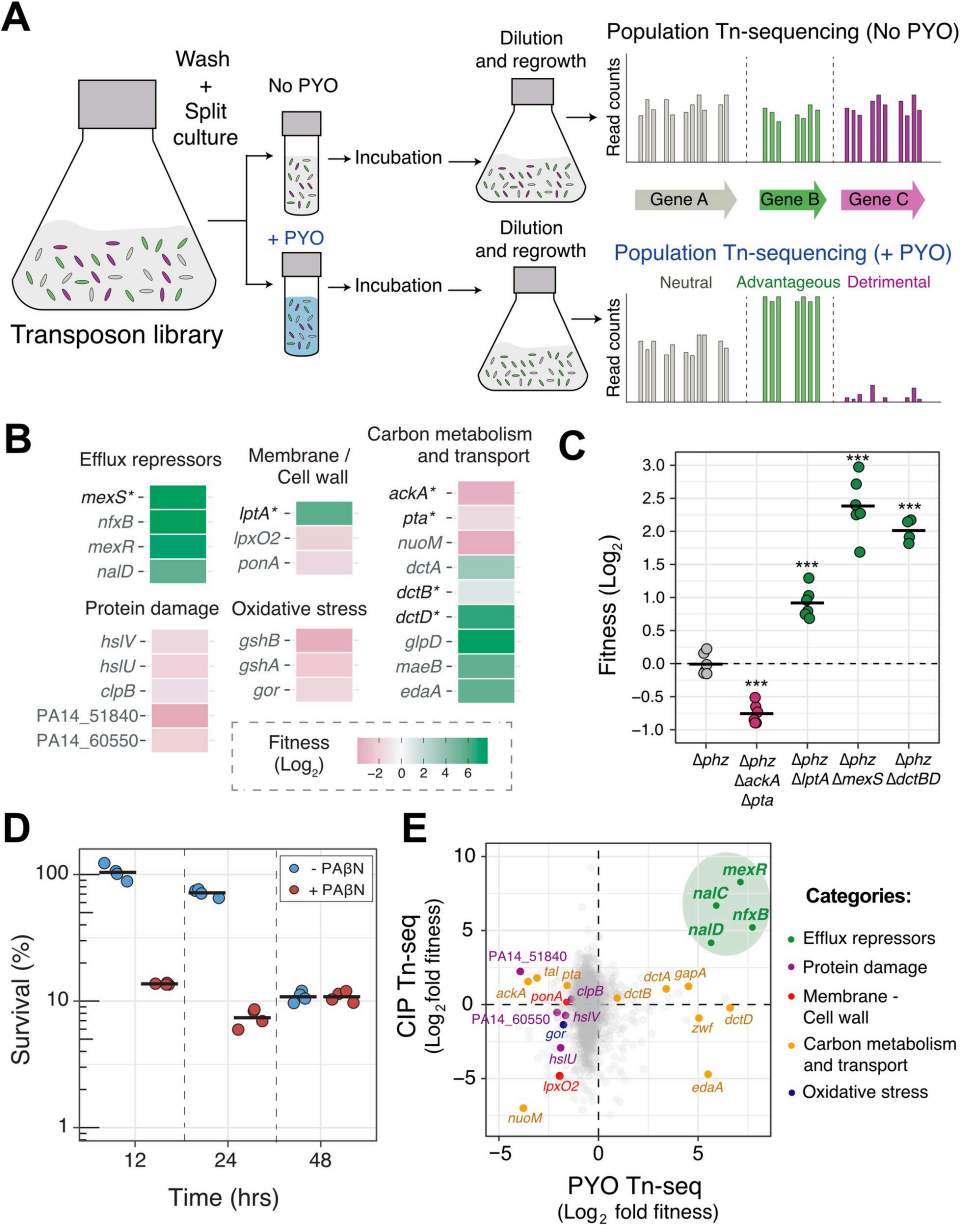
Mechanisms of tolerance to the self-produced natural antibiotic PYO in *P*. *aeruginosa*. **(A)** Genome-wide Tn-seq experimental design. Cells were incubated with and without PYO under nutrient starvation for maximum PYO toxicity [16] (see Methods for details). Bar graphs shown are hypothetical representations of the expected results for genes with different fitness effects and are not derived from the obtained data. **(B)** Statistically significant fitness effects of transposon insertions in different representative genes under conditions that maximize PYO toxicity (for full dataset, see S1 Table). See Methods for details on calculation of fitness. Asterisks show genes for which chromosomal clean deletion mutants were constructed and validated. **(C)** Tn-seq validations. Chromosomal clean deletion mutants were exposed to PYO under carbon starvation, similar to the conditions used for the Tn-seq experiment. Survival of each strain was measured by CFUs and compared to the survival of the parent Δ*phz* strain for fitness calculation (see Methods for details). Statistical significance was calculated using 1-way ANOVA with Tukey HSD multiple comparison test, with asterisks showing significant differences relative to Δ*phz* (*** *p* < 0.001). Data points represent independent replicates, and black horizontal lines mark the mean fitness for each strain. **(D)** Tolerance to PYO toxicity in the presence and absence of the efflux inhibitor PAβN. Each data point represents an independent biological replicate (*n* = 4), and the horizontal black lines mark the mean survival for each condition and time point. **(E)** Fitness correlation analysis between PYO tolerance Tn-seq (this study) and CIP persistence Tn-seq [24]. Efflux repressors present in both datasets are highlighted in green. For full analysis, see S1 Table. The data underlying this figure can be found in Table A in S1 Data. ANOVA, analysis of variance; CFU, colony-forming unit; CIP, ciprofloxacin; HSD, honestly significant difference; PAβN, phenylalanine-arginine β-naphthylamide; PYO, pyocyanin; Tn-seq, transposon sequencing.

We started by characterizing the defense mechanisms *P*. *aeruginosa* has evolved to tolerate its most toxic self-produced phenazine, pyocyanin (PYO) [10,16]. To do so in an unbiased fashion, we performed a genome-wide transposon sequencing (Tn-seq) screen in which the mutant library was exposed to PYO under starvation to maximize PYO toxicity [16], and tolerance of the pooled mutants to PYO was assessed following regrowth (Fig 1A). This revealed 5 broad categories of genes that significantly affect tolerance to PYO: (i) efflux system repressors; (ii) protein damage responses; (iii) membrane or cell wall biosynthesis; (iv) oxidative stress responses; and (v) carbon metabolism and transport (Fig 1B). We validated the screen results by constructing and testing chromosomal clean deletion mutants for four of these genes (Fig 1C).

The fitness effects of different transposon insertions largely aligned with what is thought to be the primary mode of PYO toxicity, which is the generation of reactive oxygen species (ROS) [19,20]. For example, the fact that transposon insertions in different genes within the “carbon metabolism and transport” category had opposite effects on fitness likely reflects conflicting priorities for cells challenged with ROS-generating toxins: On the one hand, limiting flux through the electron transport chain decreases the potential for ROS generation, but on the other hand, proton motive force is required to pump the toxin out, and NADH is needed to power reductases involved in repair of oxidative damage. The need to counteract oxidative stress would also explain why transposon insertions in genes related to protein damage repair and glutathione synthesis or reduction led to decreased fitness in the presence of PYO (Fig 1B). Finally, for genes related to cell wall/membrane synthesis, the transposon insertions may have altered cellular permeability and thereby either increased or decreased PYO influx.

However, the strongest hits in our Tn-seq were transposon insertions in transcriptional repressors of resistance-nodulation-division (RND) efflux system genes (Fig 1B, S1 Table), which would cause overexpression of the downstream efflux pumps. These insertions dramatically increased fitness in the presence of PYO, suggesting that one of the most effective defenses against PYO toxicity is to decrease the intracellular concentration of the toxin. While transposon insertions in the genes encoding the efflux pump proteins themselves did not have strong effects in our screen (S1 Table), this is likely due to partial functional redundancy among the various efflux systems [21]. Indeed, when we challenged starved *P*. *aeruginosa* with PYO in the presence of the broad-spectrum RND efflux inhibitor phenylalanine-arginine β-naphthylamide (PAβN), cell death was accelerated, confirming that efflux pumps are necessary for minimizing PYO toxicity (Fig 1D).

Mutations in the efflux system repressors identified in our Tn-seq screen are commonly found in clinical isolates that are resistant to synthetic fluoroquinolone antibiotics, as the efflux systems regulated by these repressors efficiently export this class of drugs [22,23]. We therefore asked whether the mechanisms used by *P*. *aeruginosa* to tolerate PYO toxicity might overlap more broadly with those that confer tolerance to fluoroquinolones. To address this question, we compared our dataset to a recent Tn-seq study that screened for genes that affect *P*. *aeruginosa* survival in the presence of the broad-spectrum fluoroquinolone ciprofloxacin [24]. Across the 2 datasets, we observed similar fitness effects for insertions in a small number of genes within the “protein damage response,” “membrane/cell wall,” and “oxidative stress response” categories, but the most dramatic fitness increases in both experiments were caused by insertions in a shared set of efflux system repressors (Fig 1E, S1 Table). These results highlighted the potential for a conserved molecular route to increased tolerance against both a natural antibiotic, PYO, and a synthetic clinical antibiotic, ciprofloxacin.

### PYO induces expression of specific efflux systems, conferring cross-tolerance to fluoroquinolones

**Fig 2:**
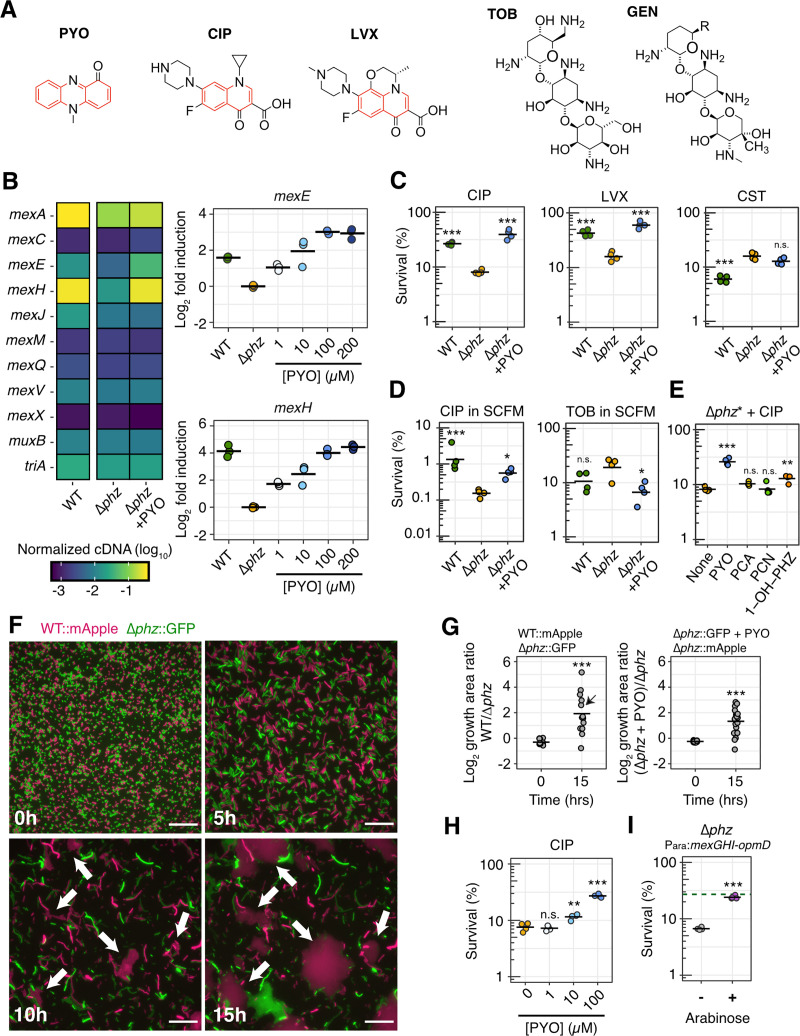
PYO induces expression of specific efflux systems, conferring cross-tolerance to fluoroquinolones. **(A)** Structures of PYO, 2 representative fluoroquinolones (CIP and LVX) and 2 representative aminoglycosides (GEN and TOB). PYO and fluoroquinolones are pumped by MexEF-OprN and MexGHI-OpmD, while aminoglycosides are not [21,22]. Rings with an aromatic character are highlighted in red. **(B)** Normalized cDNA levels for genes within operons coding for the 11 main RND efflux systems in *P*. *aeruginosa* (left; *n* = 3) and PYO-dose-dependent changes in expression of *mexEF-oprN* and *mexGHI-opmD* systems (right; *n* = 3). For full qRT-PCR dataset, see S1–S3 Figs. **(C)** Effect of PYO on tolerance to CIP (1 μg/mL), LVX (1 μg/mL), and CST (16 μg/mL) in GMM (*n* = 4). **(D)** Effect of PYO on tolerance to CIP (1 μg/mL) and TOB (40 μg/mL) in SCFM (*n* = 4). PYO itself was not toxic under the experimental conditions [16] (S4C Fig). WT made 50–80 μM PYO as measured by absorbance of the culture supernatant at 691 nm. See S5A Fig for experimental design. **(E)** Effect on tolerance to CIP (1 μg/mL) in GMM caused by the presence of the 4 main phenazines produced by *P*. *aeruginosa* (PYO, PCA, PCN, and 1-OH-PHZ) (*n* = 4). For this experiment, a Δphz* strain that cannot produce or modify any phenazine was used (see Methods). **(F, G)** Effect of PYO on lag during outgrowth after exposure to CIP in GMM. A representative field of view over different time points (F; magenta = WT::mApple, green = Δ*phz*::GFP; see S1 Movie) is shown together with the quantification of growth area on the agarose pads at time 0 hour and 15 hours (G). For these experiments, a culture of each strain tested was grown and exposed to CIP (10 μg/mL) separately, then cells of both cultures were washed, mixed, and placed together on a pad and imaged during outgrowth. The pads did not contain any PYO or CIP (see Methods and S5D Fig for details). White arrows in the displayed images point to regions with faster recovery of WT growth. The field of view displayed is marked with a black arrow in the quantification plot. The results for the experiment with swapped fluorescent proteins are shown in S4E Fig. See S4C Fig for complementary data about effects of PYO on lag. Scale bar: 20 μm. **(H)** Tolerance of Δ*phz* to CIP (1 μg/mL) in GMM in the presence of different concentrations of PYO (*n* = 4). **(G)** Tolerance of Δ*phz* to CIP (1 μg/mL) in GMM upon artificial induction of the *mexGHI-opmD* operon with arabinose (*n* = 4). The dashed green line marks the average survival of PYO-producing WT under similar conditions (without arabinose). Statistics: C, D, E, H—1-way ANOVA with Tukey HSD multiple comparison test, with asterisks showing significant differences relative to untreated Δ*phz* (no PYO); G, I—Welch unpaired *t* test (* *p* < 0.05, ** *p* < 0.01, *** *p* < 0.001, n.s. *p* > 0.05). In all panels with quantitative data, black horizontal lines mark the mean value for each condition. Individual data points represent independent biological replicates, except for in panel G, where the data points represent different fields of view. The data underlying this figure can be found in Table B in S1 Data. 1-OH-PHZ, 1-hydroxyphenazine; ANOVA, analysis of variance; CIP, ciprofloxacin; CST, colistin; GEN, gentamicin; GMM, glucose minimal medium; HSD, honestly significant difference; LVX, levofloxacin; PCA, phenazine-1-carboxylic acid; PCN, phenazine-1-carboxamide; PYO, pyocyanin; qRT-PCR, quantitative reverse transcriptase PCR; RND, resistance-nodulation-division; SCFM, synthetic cystic fibrosis sputum medium; TOB, tobramycin; WT, wild-type.

Given that cellular processes involved in PYO tolerance have also been implicated in ciprofloxacin tolerance, we asked whether exposure to PYO could promote an increase in tolerance to ciprofloxacin and related clinical antibiotics, including other synthetic fluoroquinolones. Importantly, such an effect would require that PYO induces the expression of shared defense mechanisms. We have previously established that PYO up-regulates expression of not only the oxidative stress response genes *ahpB* (an alkyl hydroperoxide reductase) and *katB* (a catalase) [16], but also at least 2 efflux systems known to pump fluoroquinolones, *mexEF-oprN* and *mexGHI-opmD* [13,16]. We confirmed these expression patterns by performing quantitative reverse transcriptase PCR (qRT-PCR) on the wild-type (WT) strain that produces PYO, a Δ*phz* mutant that does not produce PYO, and Δ*phz* treated with exogenous PYO (S1–S3 Figs). Notably, phenazines and fluoroquinolones both contain at least 1 aromatic ring, unlike other antibiotics that are not thought to be pumped by *mexEF-oprN* and *mexGHI-opmD*, such as aminoglycosides [21] (Fig 2A). Thus, structural similarities could account for why efflux pumps that likely evolved to export natural antibiotics such as PYO can also transport certain classes of synthetic antibiotics. To determine whether PYO also induces other efflux systems known to pump clinical antibiotics besides fluoroquinolones, we performed qRT-PCR on representative genes from all 11 major RND efflux systems in the *P*. *aeruginosa* genome. These measurements confirmed that *mexEF-oprN* and *mexGHI-opmD* are the only 2 efflux systems significantly induced by PYO and that the induction is PYO dose dependent (Fig 2B, S2 and S3 Figs). The *mexGHI-opmD* system in particular reached expression levels comparable to the constitutively expressed *mexAB-oprM* efflux system (Fig 2B, S2 Fig), which plays an important role in the intrinsic antibiotic tolerance and resistance of *P*. *aeruginosa* [21].

To assess whether the induction of efflux pumps and oxidative stress responses by PYO could increase the tolerance of *P*. *aeruginosa* to clinical drugs such as ciprofloxacin, we grew cultures with or without clinically relevant concentrations of PYO [25] and performed a survival assay following treatment with different antibiotics. Importantly, we hypothesized that PYO would not be a universal antagonist to all clinical antibiotics. Instead, we expected tolerance to increase only for drugs affected by the defense mechanisms induced by PYO in the cells. Indeed, compared to the non-PYO-producing Δ*phz* mutant, the PYO-producing WT strain and PYO-treated Δ*phz* were more tolerant to both ciprofloxacin and another fluoroquinolone, levofloxacin (Fig 2C). On the other hand, PYO did not confer increased tolerance to (i) aminoglycosides (Fig 2D, S4B Fig), which are not substrates for the efflux pumps up-regulated by PYO [21]; or (ii) colistin (polymyxin E) (Fig 2C), an antimicrobial peptide that permeabilizes the outer membrane of the cell by interacting with the lipopolysaccharide and causing displacement of divalent cations [1]. Similar to aminoglycosides, colistin is not known to be pumped by the PYO-induced efflux systems [21]; moreover, efflux rarely impacts polymyxin efficacy [26]. PYO itself was not toxic under the experimental conditions used in our tolerance assays [16] (S4C Fig). Aside from PYO, 1-hydroxyphenazine was the only other phenazine made by *P*. *aeruginosa* that increased tolerance to ciprofloxacin under our conditions, albeit to a lesser extent than PYO (Fig 2E). We also tested whether the presence of PYO could affect the minimum inhibitory concentration (MIC) for ciprofloxacin, as the classical definition of antibiotic tolerance also stipulates that increased survival in the presence of an antibiotic is not accompanied by an increase in MIC [2,8]. When we determined the MIC for ciprofloxacin according to standard clinical protocols [27] for our *P*. *aeruginosa* strain in the presence or absence of PYO, we saw no consistent difference at a detection limit of a 2-fold increase in MIC (S7 Table), supporting the interpretation that the effect of PYO on *P*. *aeruginosa* is primarily an increase in antibiotic tolerance (i.e., survival without the ability to grow) rather than phenotypic resistance. Importantly, PYO also induced ciprofloxacin tolerance when *P*. *aeruginosa* was grown in synthetic cystic fibrosis sputum medium (SCFM) (Fig 2D), suggesting that PYO production could contribute to antibiotic tolerance of this bacterium in CF patients. Together, these results indicate that PYO preferentially induces tolerance to fluoroquinolones.

Under in vitro conditions, PYO is typically produced in early stationary phase [13]. However, the heterogeneous nature of physiological conditions in infections [28,29] could lead to intermixing of PYO-producing and PYO-non-producing cells in vivo. We therefore tested whether exogenous PYO could increase the fluoroquinolone tolerance of cells harvested during log phase, which did not make PYO. To limit the growth of the no-antibiotic control, we exposed these cells to the antibiotics under nitrogen depletion. PYO still increased tolerance to both ciprofloxacin and levofloxacin under these conditions, suggesting that the induced tolerance phenotype does not depend on the previous growth phase of growth-arrested cells [16] (S4B Fig). Next, to visualize the recovery of cell growth after a transient exposure to ciprofloxacin, we performed a time-lapse microscopy assay (S4D Fig). Interestingly, WT *P*. *aeruginosa* and PYO-treated Δ*phz* exhibited a shorter lag phase compared to non-PYO-treated Δ*phz* following ciprofloxacin treatment (Fig 2F and 2G, S4E and S4F Fig, S1 Movie), suggesting that PYO-induced defenses may help minimize cellular damage during the antibiotic treatment. We also found that addition of PYO to Δ*phz* increased ciprofloxacin tolerance in a dose-dependent manner (Fig 2H), mirroring the dose-dependent induction of *mexEF-oprN* and *mexGHI-opmD* (Fig 2B).

Given that PYO-induced efflux pumps transport specific substrates [21], we asked if increased drug efflux could be the primary mechanism underlying PYO-mediated tolerance to fluoroquinolones. At high concentrations of ciprofloxacin, addition of the efflux inhibitor PAβN eliminated the survival advantage of PYO-treated cells, indicating that efflux pump activity is necessary for the PYO-mediated increase in antibiotic tolerance (S4G Fig). Next, we constructed a Δ*phz* strain with the *mexGHI*-*opmD* operon under the control of an arabinose-inducible promoter (P_ara_:*mexGHI*-*opmD*). We verified that the transcription levels of *mexGHI-opmD* under arabinose induction were comparable to when PYO is present (S5 Fig). Indeed, arabinose induction of *mexGHI-opmD* expression increased ciprofloxacin tolerance to near WT levels (Fig 2I), suggesting that induction of this efflux system is sufficient to confer the PYO-mediated increase in tolerance. On the other hand, arabinose induction of the oxidative stress response genes *ahpB* or *katB* did not significantly increase tolerance of Δ*phz* to ciprofloxacin (S6A and S6B Fig); however, the levels of induction achieved for these 2 genes with arabinose were lower than those observed in the presence of PYO (S1–S5 Figs). Importantly, the clinical relevance of *mexGHI-opmD* was previously not well known; as to our knowledge, there have been no reports of clinical mutants with constitutive overexpression of this efflux system. Taken together, our results demonstrate that PYO-mediated regulation of *mexGHI-opmD* expression modulates tolerance to a particular class of clinically used antibiotics in *P*. *aeruginosa*.

### PYO promotes the evolution of antibiotic resistance in *P*. *aeruginosa*

**Fig 3:**
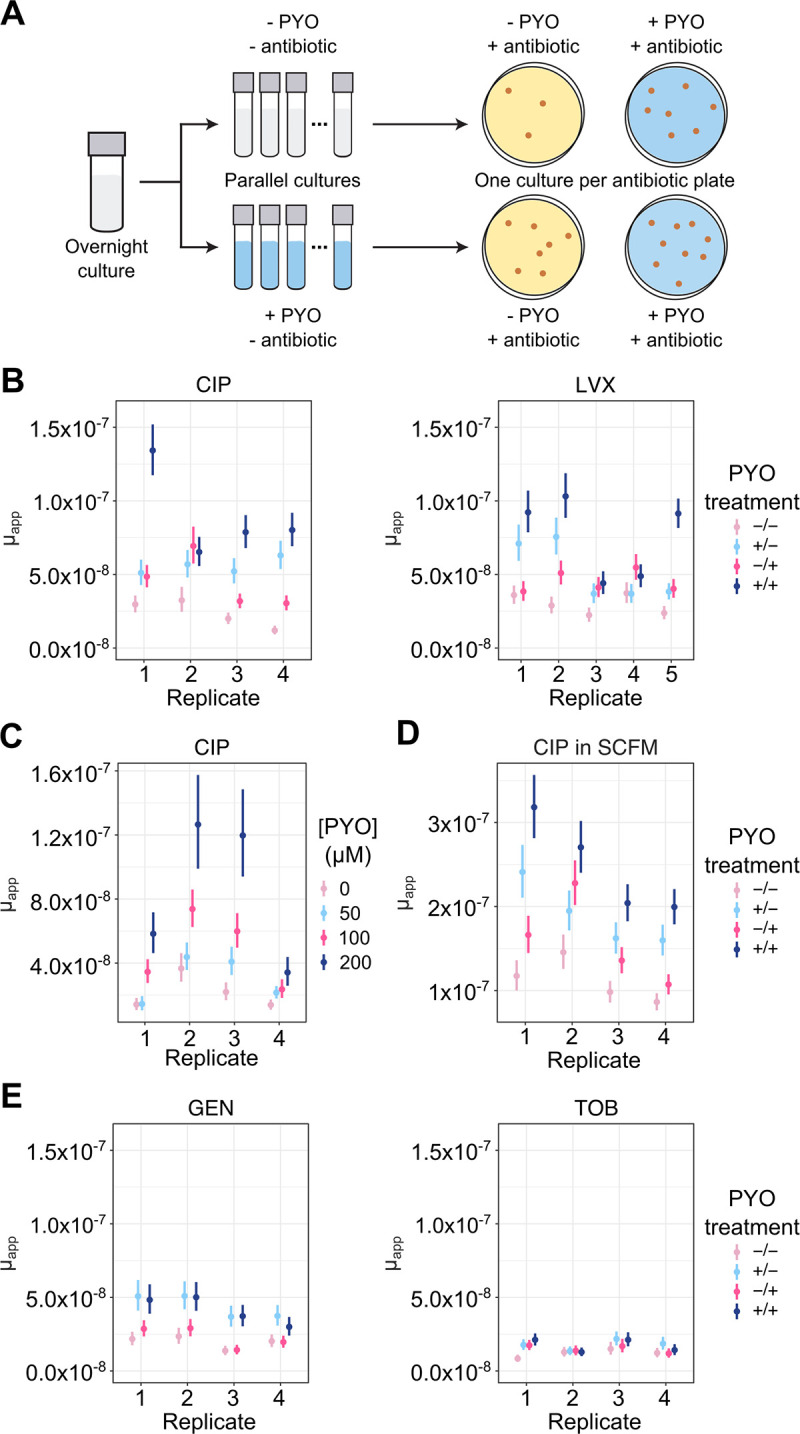
PYO increases the apparent rate of mutation to antibiotic resistance in *P*. *aeruginosa*. **(A)** Experimental design for fluctuation tests to determine the effect of PYO (100 μM unless otherwise noted) on apparent mutation rates. For panels B–E, mutation rates were calculated using an established maximum likelihood-based method that accounts for the effects of plating a small proportion of the total culture volume (see Methods for details). Each data point in those panels represents a single biological replicate comprising 44 parallel cultures, and the vertical lines represent the 84% confidence intervals. Lack of overlap in these confidence intervals corresponds to statistical significance at the *p* < 0.05 threshold [99]. For statistical significance as determined by a likelihood ratio test, see S2 Table. In B, D, and E, the PYO treatments correspond to the following: −/− denotes no PYO pretreatment (in the liquid culture stage) or co-treatment (in the antibiotic agar plates), +/− denotes PYO pretreatment but no co-treatment, −/+ denotes PYO co-treatment without pretreatment, and +/+ denotes both PYO pretreatment and co-treatment. **(B)** Apparent mutation rates of log-phase Δ*phz* grown in GMM and plated on MH agar containing CIP (0.5 μg/mL; *n* = 4) or LVX (1 μg/mL; *n* = 5), with or without pre- and/or co-exposure to PYO relative to the antibiotic selection step. **(C)** The apparent rate of mutation to resistance for Δ*phz* cells that were pretreated with different concentrations of PYO in GMM and plated onto MH agar containing CIP (0.5 μg/mL). **(D)** Apparent mutation rates of log-phase Δ*phz* grown in SCFM and plated on SCFM agar containing CIP (1 μg/mL; *n* = 4) with or without pre- and/or co-exposure to PYO. **(E)** Apparent mutation rates of log-phase Δ*phz* grown in GMM and plated during onto MH agar containing GEN (16 μg/mL; *n* = 4) or TOB (4 μg/mL; *n* = 4), with or without pre- and/or co-exposure to PYO. The data underlying this figure can be found in Table C in S1 Data. CIP, ciprofloxacin; GEN, gentamicin; GMM, glucose minimal medium; LVX, levofloxacin; MH, Mueller–Hinton; PYO, pyocyanin; SCFM, synthetic cystic fibrosis sputum medium; TOB, tobramycin.

Previous studies have demonstrated that mutations conferring antibiotic tolerance or persistence promote the evolution of antibiotic resistance [3,4]. Moreover, tolerance mutations can (i) interact synergistically with resistance mutations to increase bacterial survival during antibiotic treatment [30]; and (ii) promote the establishment of resistance mutations during combination drug therapy [31]. To assess whether antibiotic tolerance induced by PYO could similarly promote the establishment of resistance mutations in populations of *P*. *aeruginosa* undergoing extended exposure to a clinical antibiotic, we next performed a series of fluctuation tests (Fig 3A). In clinical settings, antibiotic resistance is likely to result in treatment failure if a pathogen can grow at antibiotic concentrations above a threshold commonly referred to as a “breakpoint.” We adopted this criterion by selecting mutants on antibiotic concentrations equal to or higher than the breakpoints defined by the European Committee on Antimicrobial Susceptibility Testing (EUCAST) [32]. Furthermore, we added PYO to our cultures either prior to the antibiotic selection step and/or concurrently with the antibiotic selection, in order to distinguish between the effects of preemptive versus continuous induction of PYO-regulated cellular defenses. Finally, while mutation rates inferred from fluctuation tests have sometimes been assumed to correlate with the per-base mutation rate across the genome [33,34], the results from these assays are also affected by the number of unique possible mutations that permit growth under the selection condition [35]. To encompass both possibilities in this study, we use the term μ_app_ (apparent rate of mutation) as a proxy for the likelihood of evolving antibiotic resistance. We calculated this parameter using standard methods for fluctuation test analysis (see Methods for details).

Regardless of whether PYO was added prior to or concurrently with the antibiotic selection, PYO significantly increased μ_app_ for resistance to ciprofloxacin in log-phase cultures (Fig 3B, S2 and S3 Tables). The same trends were also observed in stationary-phase cultures, albeit with smaller effect sizes (S7A Fig). These results indicate that pretreatment with PYO is sufficient but not necessary to increase μ_app_ for ciprofloxacin resistance. Adding PYO at both stages of the fluctuation test generally resulted in an even greater increase in μ_app_ (Fig 3B, S7A Fig), and the increase in μ_app_ when PYO was added prior to antibiotic selection was dose dependent (Fig 3C). Cultures that were selected on levofloxacin similarly displayed an increased μ_app_ upon PYO treatment, although the impact of pretreatment versus co-treatment with PYO varied across biological replicates (Fig 3B, S2 and S3 Tables). More importantly, PYO significantly increased μ_app_ for cultures that were grown in liquid SCFM and selected on SCFM plates containing ciprofloxacin (Fig 3D, S3 Table), suggesting that PYO produced by *P*. *aeruginosa* could promote mutation to antibiotic resistance in chronically infected lungs of CF patients [36].

Because PYO did not increase tolerance to aminoglycosides (Fig 2D, S4B Fig), we hypothesized that PYO would not promote mutation to aminoglycoside resistance if the induction of shared defense mechanisms was required for the observed increases in μ_app_. On the other hand, if PYO affected μ_app_ primarily by acting as a mutagen, pretreatment with PYO before antibiotic selection would be expected to increase μ_app_ by a similar proportion for resistance to all classes of antibiotics. To differentiate between these modes of action, we repeated the fluctuation tests using gentamicin and tobramycin, representative members of the aminoglycoside class that disrupt protein translation [1]. Cultures that were preexposed to PYO consistently exhibited significant increases in μ_app_ for gentamicin resistance (Fig 3E, S2 and S3 Tables). For tobramycin resistance, on the other hand, pretreatment with PYO only significantly increased μ_app_ in one out of 4 biological replicates (Fig 3E, S2 and S3 Tables). In addition, for both aminoglycosides, adding PYO to the antibiotic selection plates had no effect on μ_app_ in most replicates (Fig 3E, S2 and S3 Tables). These differing responses to PYO depending on the choice of clinical antibiotic suggested that the observed changes in μ_app_ were related to PYO-induced cellular defenses more so than a mutagenic effect of PYO. In fact, previous studies have suggested that gentamicin generates ROS more readily than tobramycin [37,38]. This could account for why the effect of pre-exposure to PYO on μ_app_ for resistance was greater for gentamicin than for tobramycin, given that PYO primes cells to detoxify ROS by inducing oxidative stress responses (S1 Fig). For resistance to fluoroquinolones, on the other hand, simultaneous induction of multiple defenses is likely necessary to recapitulate the increases in μ_app_ upon exposure to PYO. Overexpression of individual oxidative stress genes induced by PYO did not increase μ_app_ for ciprofloxacin resistance, while overexpression of the *mexGHI-opmD* efflux system only mildly increased μ_app_ in a subset of biological replicates (S7B Fig). Interestingly, the latter result contrasted with our finding that induction of *mexGHI-opmD* was sufficient to recapitulate PYO-mediated increases in fluoroquinolone tolerance. Together, our data suggest that while tolerance and resistance can be mechanistically interrelated, overcoming the barrier to growing in the presence of an antibiotic in some cases requires a different or broader set of defenses than is required for temporary survival under growth-arrested conditions. Nevertheless, PYO-induced defense mechanisms appear to contribute to both types of resilience to antibiotic treatment.

We envisioned at least 3 ways in which, under antibiotic selection, PYO-induced defense mechanisms could lead to the apparent increases in mutation rates: (A) by enhancing the growth of preexisting “partially resistant” mutants during exposure to the antibiotic; (B) by increasing the proportion of cells that survive and subsequently mutate to resistance while still in the presence of the antibiotic; or (C) by a combination of A and B. To distinguish between these scenarios, we implemented a 2-pronged approach. First, to explore the possibility of scenario A, we isolated and characterized several mutants from the fluctuation test plates containing ciprofloxacin. We regrew the isolates under nonselective conditions both with and without PYO treatment and calculated the percentage of colony-forming units (CFUs) that could subsequently be recovered on ciprofloxacin plates relative to nonselective plates, as a metric for each isolate’s level of resistance. We defined as “partially resistant” those isolates for which only a subset of the population could grow under the antibiotic selection without PYO treatment, as evidenced by lower CFU counts on antibiotic plates compared to nonselective plates. Second, to determine the relative likelihoods of scenario A and scenario B, we examined the fit of our fluctuation test data to different formulations of the theoretical Luria–Delbrück (LD) distribution. Specifically, we compared mathematical models that make different assumptions regarding whether mutants arise prior to or during the antibiotic selection.

**Fig 4:**
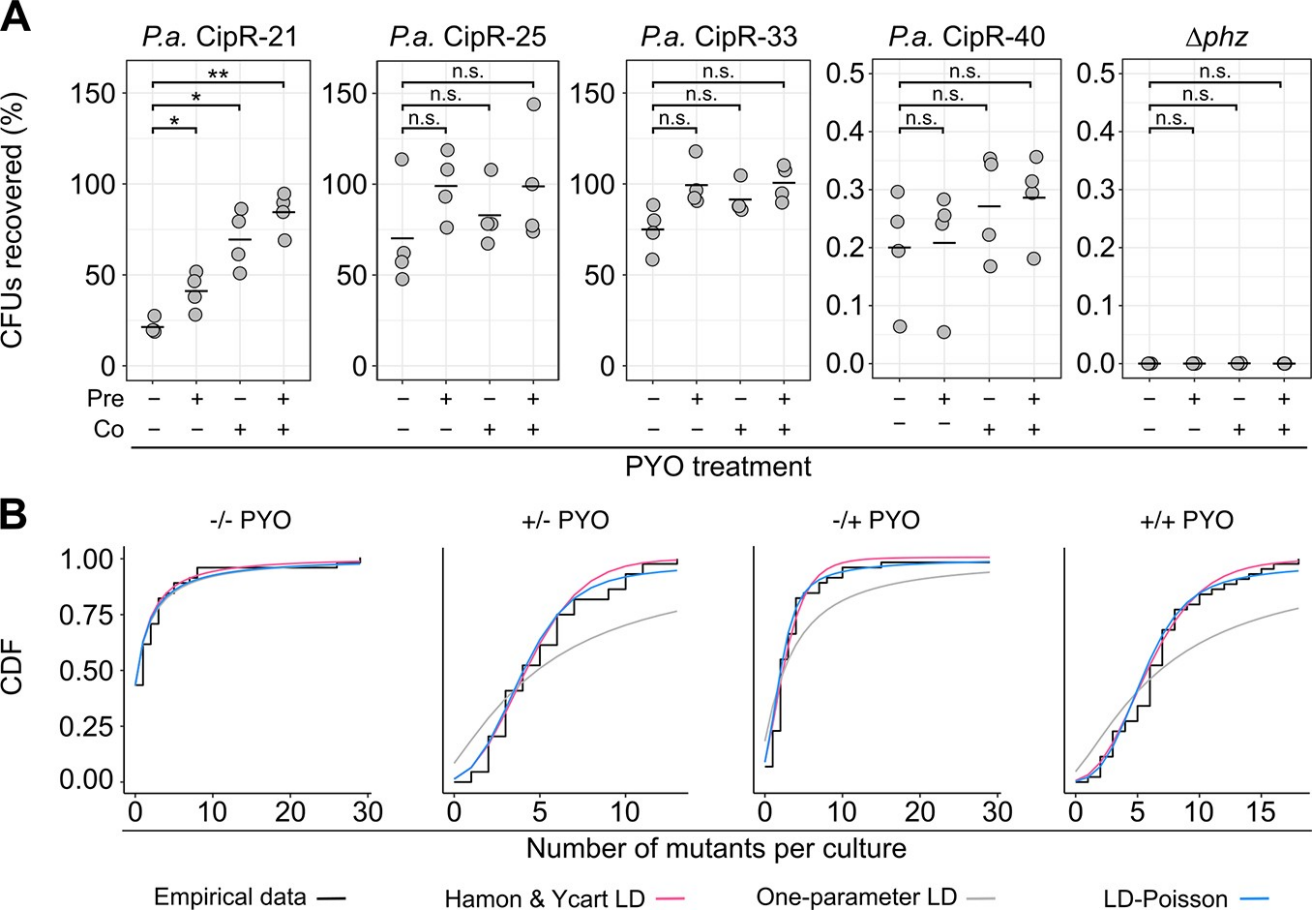
PYO promotes the growth of partially resistant mutants and the occurrence of post-plating mutations. **(A)** Putative CIP-resistant mutants of *P*.*a*. isolated from fluctuation test plates were grown to mid-log phase in liquid GMM with or without 100 μM PYO, before plating for CFUs on nonselective MH agar plates, plates containing CIP alone (0.5 μg/mL), and plates containing CIP and PYO. Plotted values represent the percentage of CFUs recovered on the CIP plates, calculated relative to total CFUs counted on nonselective plates. On the x-axis, “pre” denotes the presence of PYO in the liquid cultures, and “co” denotes the presence of PYO in the agar plates. Data points represent independent biological cultures (*n* = 4). Black horizontal lines mark the mean values for each condition. **(B)** Goodness of fit of different mathematical models for *P*. *aeruginosa* Δ*phz* fluctuation test data. Data from the fluctuation tests performed on CIP are plotted for different combinations of PYO in liquid (pretreatment) and PYO in agar (co-exposure to antibiotic selection). The empirical CDFs of the data (black) are plotted against (1) a variation of the LD model fit with 2 parameters, *m* (the expected number of mutations per culture) and *w* (the relative fitness of mutant cells vs. WT), as implemented by Hamon and Ycart [44] (pink); (2) a mixed LD and Poisson distribution fit with 2 parameters, *m* and *d* (the number of generations that occur post-plating), allowing for the possibility of post-plating mutations, as implemented by Lang and Murray [45] (blue); and (3) the basic LD distribution model fit only with *m*, as implemented by Lang and Murray [45] (gray). In each condition, the plotted experimental data represent the biological replicate with the lowest chi-squared goodness of fit *p*-value (i.e., least good fit) for the Hamon and Ycart model, demonstrating that this model was still a reasonable fit for these samples. Statistics: A—Welch unpaired *t* tests with Benjamini–Hochberg correction for controlling false discovery rate (* *p* < 0.05, ** *p* < 0.01, *** *p* < 0.001, n.s. *p* > 0.05). The data underlying this figure can be found in Table D in S1 Data. CDF, cumulative distribution function; CFU, colony-forming unit; CIP, ciprofloxacin; GMM, glucose minimal medium; LD, Luria–Delbrück; *P*.*a*., *P*. *aeruginosa*; PYO, pyocyanin; WT, wild-type.

We identified multiple partially resistant mutants for which the percentage of CFUs recovered on ciprofloxacin plates following growth under nonselective conditions increased when the isolate was either preexposed or co-exposed to PYO (Fig 4A), although the trends were not always statistically significant. Importantly, CFUs for the Δ*phz* parent strain were below the level of detection on the ciprofloxacin plates even in the presence of PYO, confirming that PYO-induced defenses alone, in the absence of a resistance mutation, were insufficient to enable growth under the selection condition used for the fluctuation tests (Fig 4A). In addition, for all characterized partially resistant mutants, the MIC for ciprofloxacin was higher than for the parent strain (S7 Table). For some of these mutants, the MIC determined according to standard clinical protocols [27] matched the ciprofloxacin concentration originally used for selection, even in the presence of PYO, but this is not surprising, as the relatively dilute inoculum (5 × 10^5^ CFU/mL) and short incubation time (18 hours) used for standard MIC assays can preclude detection of weak growth at a given antibiotic concentration. As a further validation of our assay for detection of partially resistant mutants, we also tested isolates with distinct colony morphologies that were not enriched on the PYO-containing antibiotic plates relative to PYO-free antibiotic plates in the original fluctuation tests. As expected, these mutants were fully resistant to ciprofloxacin at the original selection concentration (S8A Fig), meaning that the same number of CFUs grew on both antibiotic plates and nonselective plates even in the absence of PYO. Interestingly, the effect of PYO on ciprofloxacin resistance varied across different partially resistant isolates (Fig 4A). This suggests that PYO does not universally raise the level of resistance of the entire population, but rather interacts synergistically with specific types of mutations conferring partial resistance. Such heterogeneity could account for why the effect of PYO in the fluctuation tests varied across biological replicates, as the degree of benefit conferred by PYO would depend on the specific mutations that randomly occurred in each replicate. We also repeated the stationary-phase ciprofloxacin tolerance assay with the partially resistant isolates and found that tolerance was likewise differentially affected by PYO (S8B Fig). Interestingly, the tolerance and resistance phenotypes shared no obvious underlying pattern, again suggesting that cellular processes that affect resistance do not always equally effect tolerance and vice versa. Nevertheless, our results demonstrate that under antibiotic selection, a subset of partially resistant mutants benefits from exposure to PYO.

Whole-genome sequencing revealed that the partially resistant isolates contained mutations either in the efflux pump repressors *nfxB* or *mexS* or in genes that affected growth rate, such as a ribosomal protein, a C4-dicarboxylate transporter, and a cell wall synthesis gene (S4 Table). Mutations in *nfxB* or *mexS* were also found in the fully resistant isolates (S4 Table), albeit at different loci compared to the partially resistant isolates. Notably, *nfxB* is considered a “pathoadaptive gene” in which mutations tend to accumulate during chronic infections [39,40]. Mutations in *mexS* are less common, but have also been detected in clinical isolates [41]. Slow-growing small-colony variant mutants of *P*. *aeruginosa* have likewise been isolated from patients [42,43]. Thus, the growth benefits conferred by PYO-induced defenses during antibiotic selection could be relevant to a variety of clinically adapted strains.

That PYO increases μ_app_ at least in part by promoting the growth of preexisting partially resistant mutants was further supported by the alternative approach of evaluating the fit of our data to different mathematical models. Specifically, Pearson chi-squared test indicated that our data closely fit the Hamon and Ycart model [44] (Fig 4B, S9 Fig, S3 Table), which allows for differential fitness of mutants compared to WT cells, but assumes that all mutants arise pre-plating. However, we could not unequivocally rule out the possibility that post-plating mutations contributed to the increases in μ_app_, as a subset of our data also fit a mixed LD–Poisson model that assumes some mutations occurred during the antibiotic selection step [45] (Fig 4B, S9 Fig, S3 Table). We also performed growth curves under the culture conditions used in our fluctuation tests prior to the antibiotic selection step, with the addition of the live cell-impermeable DNA-binding dye propidium iodide (PI) as a marker for cell death. As expected from a previous study on PYO toxicity [16], cell death was undetectable prior to the sampling time point used in most of the fluctuation tests (S8D and S8E Fig). Thus, while increased population turnover due to stress can also lead to increases in μ_app_ [46], this is unlikely to underlie the effect of PYO on μ_app_. Together, these results suggest that the most probable explanation for the PYO-mediated increases in apparent mutation rates is a combined effect of increased detection of partially resistant mutants (the proposed scenario A) and increased occurrence of post-plating mutations resulting from elevated survival on the antibiotic plates (the proposed scenario B).

Importantly, previous studies based on in vitro evolution experiments have demonstrated that even modest increases in mutation rates, in the range of 2- to 5-fold, significantly affect the maximum achievable level of antibiotic resistance for diverse bacterial pathogens [47,48]. Moreover, it is well established that partial resistance can rapidly lead to acquisition of full resistance via secondary mutations [49,50]. Indeed, several putative mutants appeared fully resistant to ciprofloxacin in our CFU recovery assay despite having been enriched by exposure to PYO in the fluctuation tests (S8C Fig). This discrepancy could be a result of acquiring secondary mutations either during growth on the original fluctuation test plates or during the pre-growth for the CFU recovery assay. Thus, our results suggest that PYO may significantly affect the rate at which high-level resistance emerges in populations of *P*. *aeruginosa* undergoing long-term antibiotic exposure.

### PYO promotes antibiotic tolerance in other opportunistic pathogens

While the above experiments were performed with single species cultures, *P*. *aeruginosa* is found in polymicrobial communities in both natural environments (e.g., soil) and clinical contexts (e.g., chronic infections) [51–54]. We hypothesized that microbes that frequently interact with *P*. *aeruginosa* would have evolved inducible defense mechanisms against PYO toxicity and that production of PYO by *P*. *aeruginosa* might therefore also increase tolerance and resistance to clinical antibiotics in these community members. To test this hypothesis, we focused on the genera *Burkholderia* and *Stenotrophomonas*, both of which are (i) soil-borne gram-negative opportunistic pathogens that are frequently refractory to clinical antibiotic treatments [55–57]; and (ii) found in coinfections with *P*. *aeruginosa*, e.g., in CF patients [58]. Specifically, we tested a plant-derived strain, *B*. *cepacia* ATCC 25416; a non-CF clinical isolate of *Stenotrophomonas*, *Stenotrophomonas maltophilia* ATCC 13637; and several clinical isolates of the 3 most prevalent *Burkholderia* species found in CF patients [51]: *Burkholderia cenocepacia*, *Burkholderia multivorans*, and *Burkholderia gladioli* (for descriptions of these strains, see S5 Table).

**Fig 5:**
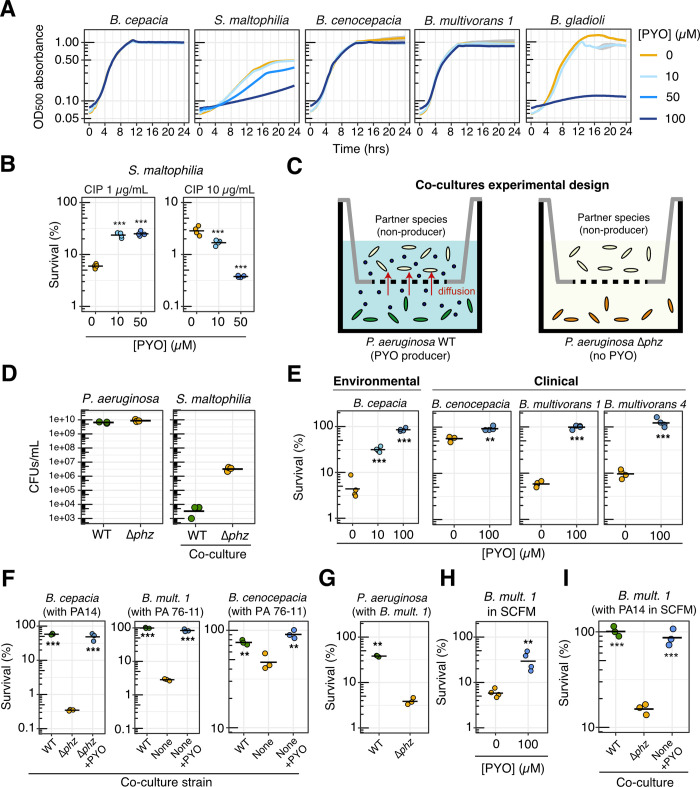
PYO promotes antibiotic tolerance in other opportunistic pathogens. **(A)** Growth in GMM + AA of several strains in the presence of different concentrations of PYO. Plotted lines represent averages of 4–6 replicates, and shaded areas in gray represent the standard deviation. *Burkholderia multivorans 1* = *B*. *multivorans* AU42096. For complete information on strains, see S5 Table. **(B)** Tolerance of *S*. *maltophilia* to different concentrations of CIP (1 or 10 μg/mL) after growth in the presence of different concentrations of PYO (0, 10, or 50 μM) in GMM + AA (*n* = 4). **(C)** Schematic depicting the experimental design for coculture antibiotic tolerance assays (see Methods for details). **(D)** CFUs recovered from cocultures of *P*. *aeruginosa* (PA14 WT and Δ*phz*) and *S*. *maltophilia* (*n* = 3), showing that the latter struggled to grow in the presence of *P*. *aeruginosa* in GMM + AA. **(E)** Effect of PYO on the tolerance to CIP (10 μg/mL) in GMM + AA of multiple *Burkholderia* species isolated from environmental and clinical samples (*n* = 4). **(F)** Effect of PYO produced by *P*. *aeruginosa* in cocultures on the tolerance of different *Burkholderia* species to CIP (10 μg/mL) in GMM + AA. PA14 is our model laboratory strain of *P*. *aeruginosa*, while PA 76–11 is a PYO-producing strain of *P*. *aeruginosa* isolated from a CF patient. The *Burkholderia* strains were plated separately for CFUs to assess survival following treatment with CIP in the cocultures (*n* = 3). **(G)** Tolerance of *P*. *aeruginosa* PA14 WT and Δ*phz* to CIP (1 μg/mL) when grown in cocultures with *B*. *multivorans 1* in GMM + AA (*n* = 3). **(H)** Effect of PYO on the tolerance to CIP (10 μg/mL) of *B*. *multivorans 1* in SCFM (*n* = 4). **(I)** Tolerance to CIP (1 μg/mL) in SCFM of *B*. *multivorans 1* grown in cocultures with P. *aeruginosa* PA14 WT, Δ*phz*, or alone with 100 μM PYO added exogenously (*n* = 3). Statistics: B, E, F, G, H, I—1-way ANOVA with Tukey HSD multiple comparison test for comparisons of 3 conditions or Welch unpaired *t* test for comparison of 2 conditions, with asterisks showing the statistical significance of comparisons with the untreated (no PYO or Δ*phz*) condition (* *p* < 0.05, ** *p* < 0.01, *** *p* < 0.001). In all panels, data points represent independent biological replicates, and black horizontal bars mark the mean values for each condition. The data underlying this figure can be found in Table E in S1 Data. AA, amino acids; ANOVA, analysis of variance; CF, cystic fibrosis; CFU, colony-forming unit; CIP, ciprofloxacin; GMM, glucose minimal medium; HSD, honestly significant difference; PYO, pyocyanin; SCFM, synthetic cystic fibrosis sputum medium; WT, wild-type.

We first assessed each strain’s intrinsic resistance to PYO (Fig 5A), as we expected that strong defenses against PYO toxicity would be required in order to benefit from exposure to this natural antibiotic. Indeed, for *S*. *maltophilia*, which was sensitive to PYO (Fig 5A), the effects of PYO on antibiotic tolerance were complex: The presence of PYO was only beneficial when ciprofloxacin levels were low (1 μg/mL) (Fig 5B). At a higher concentration of ciprofloxacin (10 μg/mL), PYO was detrimental in a dose-dependent manner (Fig 5B), suggesting that the additional stress conferred by PYO outweighed any induction of defense mechanisms against ciprofloxacin. *S*. *maltophilia* also struggled to grow with *P*. *aeruginosa* in cocultures (Fig 5C and 5D), indicating that the conditions under which this species could potentially benefit from PYO are very limited.

*B*. *cepacia*, *B*. *cenocepacia*, and *B*. *multivorans*, on the other hand, were highly resistant to PYO (Fig 5A). For these 3 species, exogenously added PYO increased tolerance to ciprofloxacin (Fig 5E). Furthermore, for *B*. *cepacia*, we confirmed that this effect was PYO dose dependent (Fig 5E). We therefore tested whether *P*. *aeruginosa* could induce tolerance to ciprofloxacin in cocultures with these *Burkholderia* strains. Using liquid culture plates in which the 2 species were separated by a permeable membrane (Fig 5C), we found that PYO-producing *P*. *aeruginosa* strongly induced tolerance to ciprofloxacin in the *Burkholderia* species and that the observed tolerance phenotypes were recapitulated by addition of exogenous PYO to cocultures of *Burkholderia* and the *P*. *aeruginosa* Δ*phz* mutant or to control cultures with *Burkholderia* alone in the same setup (Fig 5F). Notably, for *B*. *cenocepacia* and *B*. *multivorans*, increased ciprofloxacin tolerance was also observed in cocultures with a PYO-producing strain isolated from a CF patient, *P*. *aeruginosa* PA 76–11 (Fig 5F). In addition, even when cocultured with *Burkholderia*, the *P*. *aeruginosa* WT strain still showed elevated ciprofloxacin tolerance when compared to the non-PYO-producing Δ*phz* mutant (Fig 5G). This indicates that the presence of *Burkholderia* did not alter *P*. *aeruginosa* tolerance patterns under our conditions. Finally, similar results were obtained for experiments performed in SCFM, where either the addition of exogenous PYO (Fig 5H) or coculture with *P*. *aeruginosa* (Fig 5I) led to increased tolerance levels in *Burkholderia*. Together, these results suggest that PYO produced by *P*. *aeruginosa* in CF patients may decrease the efficacy of ciprofloxacin as a treatment for multispecies infections.

### PYO promotes the evolution of antibiotic resistance in a co-occurring opportunistic pathogen

**Fig 6:**
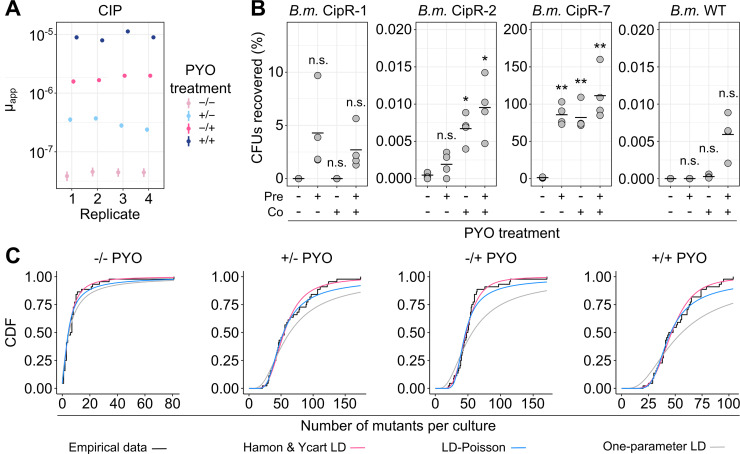
PYO promotes antibiotic resistance in *B*. *multivorans*. **(A)** The apparent rate of mutation to resistance when log-phase *B*. *multivorans 1* cells grown in GMM + AA were plated on MH agar containing CIP (8 μg/mL), with or without pre- and/or co-exposure to 100 μM PYO relative to the antibiotic selection step. Each data point represents a biological replicate comprising 44 parallel cultures (*n* = 4). The vertical lines represent 84% confidence intervals, in which lack of overlap corresponds to statistical significance at the *p* < 0.05 level [99]. The PYO treatments correspond to the following: −/− denotes no PYO pretreatment (in the liquid culture stage) or co-treatment (in the antibiotic agar plates), +/− denotes PYO pretreatment but no co-treatment, −/+ denotes PYO co-treatment without pretreatment, and +/+ denotes both PYO pretreatment and co-treatment. **(B)** The percentage of CFUs recovered on CIP plates either with or without PYO in the agar, for exponential phase cultures of different partially resistant *B*. *multivorans 1* (*B*.*m*.) mutants that were pre-grown with or without PYO in liquid cultures in GMM + AA. Plotted values represent the percentage of CFUs recovered on the CIP plates, calculated relative to total CFUs counted on nonselective plates. On the x-axis, “pre” denotes the presence of PYO in the liquid cultures, and “co” denotes the presence of PYO in the agar plates. Data points represent independent biological replicates (*n* = 4), and black horizontal bars mark the mean values for each condition. **(C)** Goodness of fit of different mathematical models for *B*. *multivorans 1* fluctuation test data. Data are plotted for different combinations of PYO in liquid (pretreatment) and PYO in agar (co-exposure to antibiotic selection). The empirical CDFs of the data (black) are plotted against (1) a variation of the LD model fit with 2 parameters, *m* (the expected number of mutations per culture) and *w* (the relative fitness of mutant cells vs. WT), as implemented by Hamon and Ycart [44] (pink); (2) a mixed LD and Poisson distribution fit with 2 parameters, *m* and *d* (the number of generations that occur post-plating), allowing for the possibility of post-plating mutations, as implemented by Lang and Murray [45] (blue); and (3) the basic LD distribution model fit only with *m*, as implemented by Lang and Murray [45] (gray). In each condition, the plotted experimental data represent the biological replicate with the lowest chi-squared goodness of fit *p*-value (i.e., least good fit) for the Hamon and Ycart model. Statistics: B—Welch unpaired *t* tests with Benjamini–Hochberg correction for controlling false discovery rate (* *p* < 0.05, ** *p* < 0.01, *** *p* < 0.001). The data underlying this figure can be found in Table F in S1 Data. AA, amino acids; CDF, cumulative distribution function; CFU, colony-forming unit; CIP, ciprofloxacin; GMM, glucose minimal medium; LD, Luria–Delbrück; MH, Mueller–Hinton; PYO, pyocyanin; WT, wild-type.

We next asked whether PYO could mediate an increase in apparent mutation rate for ciprofloxacin resistance in *Burkholderia* species. We chose *B*. *multivorans* AU42096 (*B*. *multivorans* 1 in Fig 5) as our model strain for these experiments because, among the clinical isolates, it displayed the strongest response to PYO in the ciprofloxacin tolerance assays. Remarkably, when selecting *B*. *multivorans* mutants on ciprofloxacin, we observed PYO-mediated increases in μ_app_ that were far more dramatic than for *P*. *aeruginosa*: Pretreatment with PYO increased μ_app_ for *B*. *multivorans* approximately 10-fold, while co-exposure to PYO in the antibiotic plate without pre-exposure increased μ_app_ approximately 40-fold, and the combination of pre- and co-exposure to PYO increased μ_app_ by 230-fold (Fig 6A, S3 Table). Notably, the magnitude of the latter effect is on par with observed differences between hypermutators, such as mutants deficient in the mismatch repair pathway, and their respective parent strains [59–61]. Moreover, hypermutators of *Burkholderia* isolated from CF infections are associated with clinical ciprofloxacin resistance [59]. In light of these observations, our results suggest that PYO could significantly affect clinical outcomes for coinfections of *P*. *aeruginosa* and *B*. *multivorans* treated with ciprofloxacin.

To verify that the *B*. *multivorans* colonies growing on ciprofloxacin in the presence of PYO were mutants, and to assess their responses to PYO, we isolated several putative mutants from the fluctuation test antibiotic plates and tested three in our CFU recovery assay. All three displayed unique profiles of ciprofloxacin resistance in response to PYO treatment, as well as different maximal levels of resistance. However, all were more resistant than the WT parent strain in the presence of PYO, and none were noticeably resistant to ciprofloxacin in this assay without exposure to PYO (Fig 6B). MIC tests performed according to clinical standards revealed that the MIC of CipR-1 was indistinguishable from that of the parent strain, while the MIC of CipR-2 was 2-fold higher than that of the parent strain in the absence of PYO but identical in the presence of PYO (S7 Table), reflecting the limitations of standard 2-fold antibiotic dilution series for revealing mild increases in resistance. The MIC of CipR-7, on the other hand, was 8-fold higher than that of the parent strain, although in the absence of PYO, the MIC of this mutant was still below the ciprofloxacin concentration used in the fluctuation tests. Notably, for all tested *B*. *multivorans* isolates, including the WT parent, the addition of PYO to the standard MIC tests increased the MIC for ciprofloxacin by 4- to 8-fold (S7 Table); however, even in the presence of PYO, the MIC for the parent strain was less than half of the ciprofloxacin concentration used in the tolerance assays, indicating that the observed tolerance phenotype for this strain cannot be fully explained by phenotypic resistance. Interestingly, the percentage of the parent strain population that could grow on ciprofloxacin in the presence of PYO (Fig 6B) was approximately equal to what would have been expected from the frequency of colonies detected in the fluctuation tests; moreover, when the CFU recovery assay was performed for the parent strain, the colonies that grew on ciprofloxacin in the presence of PYO exhibited diverse morphologies. This suggests that much of the parent strain growth on ciprofloxacin in the presence of PYO may have in fact reflected the growth of high-frequency spontaneous mutants, rather than background growth of the parent strain itself.

Whole-genome sequencing of the fluctuation test isolates revealed that *B*. *multivorans* CipR-1 possessed mutations in 3 uncharacterized regulatory genes (S6 Table). *B*. *multivorans* CipR-2 possessed mutations in 2 different homologs of the SpoT/RelA (p)ppGpp synthetase gene, which is known to affect antibiotic tolerance and resistance [62]. Finally, *B*. *multivorans* CipR-7 possessed a point mutation in DNA gyrase A (S83R), along with a point mutation in a malto-oligosyltrehalose synthase. Given that DNA gyrase A is the target of ciprofloxacin and that the specific mutated residue is likely homologous to the T83 residue that was mutated in a study of fluoroquinolone-resistant mutants in *B*. *cepacia* [63], it is intriguing that this mutant was not able to grow on the original selection concentration of ciprofloxacin in the absence of PYO; however, the specific amino acid (AA) substitution in this strain may have resulted in only a mild disruption of ciprofloxacin binding.

Lastly, we asked whether the *B*. *multivorans* mutants we detected primarily arose prior to or during the antibiotic selection. In all cases, the distribution of mutants closely matched the Hamon and Ycart formulation of the theoretical LD distribution, suggesting that the detected mutants arose prior to the antibiotic exposure (Fig 6C, S3 Table). Interestingly, the Hamon and Ycart model also predicted the average relative fitness of mutants detected in PYO-treated samples to be significantly lower compared to mutants detected in non-PYO-treated samples (S3 Table; *p* < 0.05 for all 3 comparisons between non-PYO-treated and PYO-treated sample groups, using Welch paired *t* test with Benjamini–Hochberg corrections for controlling the false discovery rate). In addition, unlike for *P*. *aeruginosa*, the mixed LD–Poisson distribution that allows for post-plating mutations was a poorer fit than the Hamon and Ycart model for all PYO-treated *B*. *multivorans* samples (Fig 6C, S3 Table). Together, these results suggest that in *B*. *multivorans*, PYO increases μ_app_ by promoting growth of a wider range of mutants that arise prior to antibiotic selection, including those with slower growth rates.

## Discussion

Many clinical antibiotic resistance genes are thought to have originated in environmental microorganisms as responses to microbial chemical warfare, with subsequent mobilization into human pathogens via horizontal gene transfer [5,6,64]. Here, we have demonstrated that tolerance and resistance to clinically relevant concentrations of synthetic antibiotics can also arise as a collateral benefit of natural antibiotic production by an opportunistic pathogen. *P*. *aeruginosa* is a particularly relevant example of an opportunistic pathogen whose self-produced natural antibiotics can promote resilience to clinical antibiotics, given the large number of chronic infections caused by this bacterium worldwide [9] and the fact that PYO has been detected at concentrations up to 130 μM in lung infection sputum samples [25] and 0.31 mg/g in infected wound exudate [65]. Notably, treatments for infections caused by *P*. *aeruginosa* and other opportunistic pathogens often fail even when in vitro MIC tests indicate susceptibility to the chosen antibiotic [58]. Previous studies have attributed this discrepancy to metabolic and physiological changes within biofilms [66,67], which represent a major form of bacterial life within infections [68]. Our results suggest that cellular defenses induced by bacterially produced natural antibiotics may also contribute to in vitro versus in vivo differences in antibiotic susceptibility, as standard MIC tests are inoculated at a low cell density [32], but *P*. *aeruginosa* typically does not make PYO in vitro until reaching a relatively high cell density [13]. Furthermore, the observation that PYO produced by *P*. *aeruginosa* strongly promotes antibiotic tolerance and resistance in *Burkholderia* species could hold important ramifications for the treatment of coinfections of these organisms in CF patients, for which clear best practices have yet to be established [58]. In particular, it could be prudent to avoid treating such infections with antibiotics for which PYO is likely to promote increased tolerance and resistance, such as fluoroquinolones, chloramphenicol, and trimethoprim/sulfamethoxazole—the latter two also being known substrates for efflux pumps that we have shown are up-regulated by PYO [9,21].

Interestingly, our finding that PYO does not increase tolerance to aminoglycosides (Fig 2D, S4B Fig) contrasts with the conclusions of 2 previous studies on phenazine-mediated antibiotic tolerance, which claimed that phenazines broadly increase tolerance to all classes of antibiotics except cationic peptides [17,18]. Importantly, however, these studies did not explore whether or which molecular defense mechanisms are induced by the phenazines and how these defenses might interact with clinical antibiotics. Moreover, the studies were performed under very different experimental setups, including different media, which can profoundly impact the outcomes of antibiotic susceptibility assays. One study focused on colony biofilms of *P*. *aeruginosa* that produced only phenazine-1-carboxylic acid (PCA) and phenazine-1-carboxamide [17], which are less toxic than PYO [16] and consequently may induce a different set of cellular responses. Alternatively, the observed increased tolerance to tobramycin in that study might not be related to molecular defenses induced by phenazines, but rather phenazine-mediated physiological differences under the studied conditions [17]. Phenazines are redox-active molecules that can promote metabolic activity under oxygen limitation, which occurs within biofilms [12,17,69]; the specific details of how such metabolic activity might affect antibiotic tolerance merit further attention. The other previous study found that PYO increased planktonic culture cell densities in the presence of various antibiotics [18], but these experiments did not directly demonstrate an effect on antibiotic tolerance (i.e., the ability to survive an otherwise lethal antibiotic treatment) [2]. Notably, both studies found that phenazines actually increase sensitivity to cationic peptides, consistent with our observation that WT *P*. *aeruginosa* is less tolerant to colistin than the *Δphz* strain. The mechanism of this synergistic lethality warrants further investigation. Our results highlight that identifying the cellular defenses induced by natural antibiotics, not only in the case of *P*. *aeruginosa* and phenazines, but also potentially other opportunistic pathogens and their endogenously produced natural antibiotics, is essential for accurately predicting clinical antibiotic efficacy.

**Fig 7:**
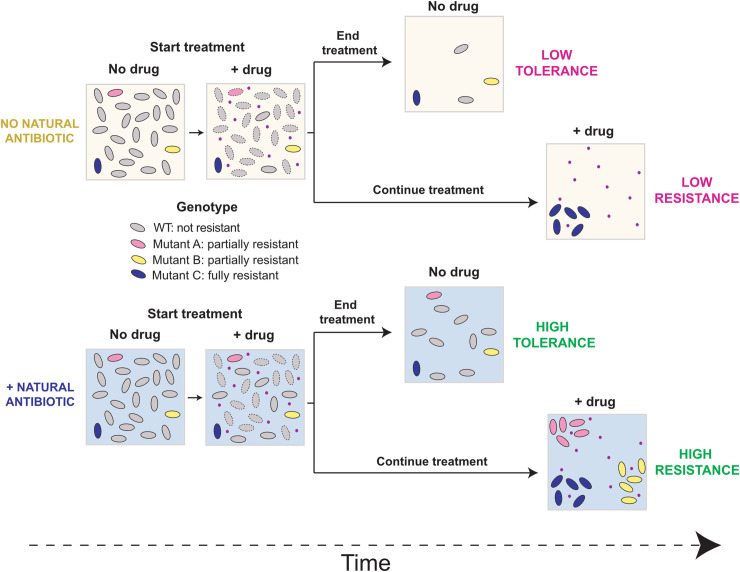
Proposed model for how natural antibiotics increase bacterial tolerance and resistance to clinical drugs. In the first scenario (tolerance), cells are exposed to the clinical drug (pink dots) for a short period of time. Surviving cells will eventually restart growth after the drug is removed. The presence of the natural antibiotic (bottom) increases tolerance of both WT and partially resistant mutants. In the second scenario (resistance), cells are constantly exposed to the drug for an extended period of time, and only mutants are maintained in the population. The presence of the natural antibiotic (bottom) widens the population bottleneck and allows partially resistant mutants to grow under drug selection, preserving a greater range of genetic diversity in the population. WT, wild-type.

Our results furthermore suggest that by inducing cellular defenses against specific clinical antibiotics, PYO widens the population bottleneck that occurs during antibiotic selection. This effect occurs via a 2-pronged mechanism (Fig 7). First, PYO increases the proportion of cells that survive short-term antibiotic treatments, which would inherently tend to preserve a greater range of genetic variation in the post-selection population. Second, PYO promotes the establishment of a broader range of resistant mutant lineages, which are likely primed to acquire further stepwise mutations to high-level resistance, yet may otherwise be lost during extended antibiotic treatment. Interestingly, a recent study demonstrated that lineages of spontaneous resistant mutants can be lost through stochastic cell death even at antibiotic concentrations well below the mutants’ MICs [70]. Thus, besides boosting the growth of partially resistant mutants whose MICs failed to exceed the antibiotic concentrations used in our fluctuation tests, it is possible that PYO-induced defenses (e.g., enhanced efflux and oxidative stress responses) also increased apparent mutation rates by decreasing the stochastic loss of individual spontaneous mutants with higher MICs. In addition, a recent study in *Staphylococcus aureus* highlighted the important role that preexisting genetic diversity in the population can play in shaping the evolution of antibiotic resistance [71]. In particular, even small variations in efflux-mediated intrinsic resistance of parent strains significantly affected the probability that a population would evolve resistance under ciprofloxacin selection at the clinical breakpoint concentration [71]. Multiple studies have also demonstrated additive or synergistic interactions between increased drug efflux and classical ciprofloxacin resistance mutations [71–73]. We observed a similar phenomenon in our *B*. *multivorans* CipR-7 strain, which had acquired a mutation in the cellular target of ciprofloxacin (i.e., DNA gyrase A), yet still required exposure to PYO in order to grow at the ciprofloxacin concentration on which it was originally selected (Fig 6B). Together, these findings suggest that microbial production of natural antibiotics in the context of an infection could dynamically, and in some cases dramatically, affect the evolvability of opportunistic pathogens challenged with clinical drugs.

Beyond *P*. *aeruginosa* and PYO, our proposed model for collateral benefits of exposure to natural antibiotics (Fig 7) potentially represents a broader phenomenon among human pathogens than has previously been appreciated. Many opportunistic pathogens originate in environments like soil [55,74], where they have evolved in the presence of diverse natural antibiotics [5,6], and *P*. *aeruginosa* is not the only pathogen with the capacity to synthesize its own antibiotics. For example, *Burkholderia* species possess the biosynthetic capability to produce a variety of compounds with antibacterial activity, whose potential clinical significance has not been explored [75]. If a given natural antibiotic induces expression of a molecular defense, the only requirement for a consequent increase in tolerance to a clinically relevant drug would be that the induced defense has some efficacy against the drug—e.g., due to structural similarities like those shared by PYO and fluoroquinolones. This inference is supported by recent evidence that certain food additives and synthetic drugs antagonize the efficacy of specific clinical antibiotics by triggering stress responses in cells, including the induction of efflux pumps [76] and that exposure to a clinical drug to which a strain is already resistant can collaterally affect its development of tolerance and resistance to other drugs [77]. In fact, bacterially produced toxic metabolites that promote antibiotic tolerance and resistance in human pathogens need not be limited to the types of molecules traditionally thought of as natural antibiotics. For example, indole secretion by highly antibiotic-resistant spontaneous mutants of *Escherichia coli* enables partially resistant mutants within the same species to grow at drug concentrations above their own MICs, in part by stimulating efflux pump expression [78]. Unlike PYO, indole is generally thought of as a signaling molecule rather than a natural antibiotic [79], although it can likewise be toxic to bacteria at high concentrations [79,80]. Efforts to identify and characterize additional examples of such metabolites produced by opportunistic human pathogens could lead to an improved understanding of the modes of antibiotic treatment failure in clinics and ultimately inform the design of more effective and longer-lived therapies.

## Methods

### Culture media and incubation conditions

Different culture media were used for different experiments as indicated throughout the Methods. Succinate minimal medium (SMM) composition was 40 mM sodium succinate (or 20 mM, if specified), 50 mM KH_2_PO_4_/K_2_HPO_4_ (pH 7), 42.8 mM NaCl, 1 mM MgSO_4_, 9.35 mM NH_4_Cl, and a trace elements solution [81]. Glucose minimal medium (GMM) was identical to SMM, except with 10 or 20 mM glucose (as specified for different experiments) instead of succinate. SMM and GMM were prepared by autoclaving all components together for 20 minutes at 121°C, except for the carbon source and the 1,000× trace elements stock solution, which were filter-sterilized and added separately; interestingly, we found that autoclaving MgSO_4_ with the other components was crucial for consistent PYO production by WT *P*. *aeruginosa* UCBPP-PA14 in GMM. Luria–Bertani (LB) Miller broth (BD Biosciences, San Jose, California, United States of America) and BBL cation-adjusted Mueller–Hinton (MH) II broth (BD Biosciences) were prepared according to the manufacturer’s instructions (notably with only a 10 minute autoclave step for MH medium), with the addition of 1.5% Bacto agar (BD Biosciences) to make solid media. SCFM composition was prepared as described previously [36], with 1.355 mM K_2_SO_4_ and no nitrate. In addition, 3.6 μM FeSO_4_.7H_2_O and 0.3 mM N-acetyl-glucosamine were added [82]. All components except for the latter two were dissolved together, sterilized by filtration through a 0.22 μm membrane, and stored for up to 2 weeks; FeSO_4_.7H_2_O and N-acetyl-glucosamine solutions were prepared fresh each time or stored at −20°C, respectively, and added to SCFM on the day of use. For SCFM agar, a 2× solution of the medium components was prepared and added to a separately autoclaved 3% molten agar solution, for a final concentration of 1× SCFM and 1.5% agar.

Antibiotics were prepared in concentrated stock solutions (100× or greater) and stored at −20°C. Ciprofloxacin was dissolved in 0.1 M or 20 mM HCl, while levofloxacin, gentamicin, tobramycin, and colistin were dissolved in sterile deionized water. PAβN dihydrochloride (MedChemExpress, Monmouth Junction, New Jersey, USA) was dissolved in sterile deionized water (50 mg/mL). PYO was synthesized and purified as previously described [83,84] and dissolved in 20 mM HCl to make 10 mM stock solutions. Experiments involving exogenous PYO always included negative controls to which an equivalent volume of 20 mM HCl was added. In addition, MH agar plates were buffered to pH 7 with 10 mM 4-morpholinepropanesulfonic acid (MOPS) to avoid any pH changes upon addition of PYO or HCl; all other media used with exogenous PYO were already inherently buffered. Incubations were always done at 37°C, with shaking for liquid cultures (250 rpm), unless mentioned otherwise.

### Strain construction

In this study, we use PA14 as an abbreviation for UCBPP-PA14. *P*. *aeruginosa* PA14 was used for all experiments unless otherwise noted. For a full list of strains made in this study, see S5 Table. Three types of strains were made in different *P*. *aeruginosa* PA14 backgrounds: (i) unmarked deletions, used for Tn-seq validation experiments; (ii) fluorescent strains for time-lapse microscopy experiments; and (iii) strains overexpressing one of the following 3 systems: *mexGHI-opmD*, *ahpB*, and *katB*. Established protocols were used for all these procedures [85].

Briefly, for unmarked deletions, approximately 1-kb fragments immediately upstream and downstream of the target locus were cloned using Gibson assembly into the pMQ30 suicide vector [86,87]. Fragments amplified from *P*. *aeruginosa* PA14 genomic DNA (gDNA) and cleaned up using the Monarch PCR Purification kit (New England Biolabs, Ipswich, Massachusetts, USA) were used for Gibson assembly together with pMQ30 cut with SacI and HindIII. The assembled construct was then transformed into *E*. *coli* DH10B, with transformants being selected in LB with 20 μg/mL gentamicin. All correctly assembled plasmids were identified by colony PCR and verified by Sanger sequencing (Laragen, Culver City, California, USA). Next, for the insertion of the constructs into *P*. *aeruginosa* PA14 genome, triparental conjugation was performed following Choi and Schweizer [88]. All unmarked deletions were done in the *P*. *aeruginosa* PA14 Δ*phz* background (both *phzA-G1* and *phzA-G2* operons are deleted in this strain [13]), allowing clean experiments by addition of exogenous phenazines. Merodiploids containing the construct integrated into their genomes were selected on VBMM medium (3 g/L trisodium citrate, 2 g/L citric acid, 10 g/L K_2_HPO_4_, 3.5 g/L NaNH_4_PO_4_·4H_2_O, 1 mM MgSO_4_, 100 μM CaCL_2_, pH 7) with 100 μg/mL gentamicin following Choi and Schweizer [88]. Finally, merodiploids were then plated on LB lacking NaCl and containing 10% sucrose to select for colonies resulting from homologous recombination. Colonies missing the target locus (unmarked deletions) were identified by PCR. For all primers used, see S5 Table.

Fluorescent strains used in time-lapse microscopy were made using previously published plasmids [85,89]. Constructs containing GFP and mApple florescent proteins under the control of the ribosomal *rpsG* gene were inserted in the *att*Tn7 site of *P*. *aeruginosa* PA14 Δ*phz* chromosome by tetraparental conjugation, followed with selection on VBMM with 100 μg/mL gentamicin [88].

Finally, overexpressing strains were made as previously described [85]. The previously made overexpression construct (pUC18T-miniTn7T-GmR vector containing the arabinose-inducible promoter P_ara_ [85]) and the 3 different targets (*mexGHI-opmD*, *ahpB*, and *katB*) were all amplified by PCR. Next, using Gibson assembly, the targets were cloned downstream of P_ara_ in the pUC18T-miniTn7T-GmR vector, resulting in the 3 different overexpression constructs: P_ara_:*mexGHI-opmD*, P_ara_:*ahpB*, and P_ara_:*katB*. The final constructs were introduced into the *att*Tn7 of the *P*. *aeruginosa* PA14 Δ*phz* background strain by tetraparental conjugation [88].

### Transposon sequencing (Tn-seq) experiment

The Tn-seq experiment was performed following the design presented in Fig 1A. Two aliquots of the *P*. *aeruginosa* PA14 transposon library previously prepared [89] were thawed on ice for 15 minutes, diluted to a starting optical density (OD_500_) of 0.05 in 50 mL of SMM, and grown aerobically under shaking conditions (250 rpm) at 37°C for approximately 4 to 5 generations to an OD_500_ of 0.8–1. These growing conditions were used for all the stages of the experiment. After growth in SMM, each aliquot was considered an independent replicate. Cells from each replicate were pelleted, washed, and resuspended (5 mL in 18 × 150 mm glass tubes, OD_500_ = 2) in minimal phosphate buffer (MPB; 50 mM KH_2_PO_4_/K_2_HPO_4_[pH 7], 42.8 mM NaCl) with and without 100 μM PYO. Cells were then incubated for 26 hours under shaking conditions at 37°C. Therefore, the experiment consisted of 4 different samples that were later sequenced: (i) “R1 No PYO”; (ii) “R1 + PYO”; (iii) “R2 No PYO”; and (iv) “R2 + PYO.” After the incubation, cultures from all treatments were pelleted, washed again to remove PYO, and resuspended in fresh SMM. Immediately, an aliquot of each sample was diluted to a starting OD_500_ of approximately 0.05 in 25 mL SMM, followed by outgrowth for approximately 4 to 5 generations to an OD_500_ of 0.8–1. After outgrowth, 2.5 mL of each sample was pelleted and stored at −80°C.

gDNA was extracted from the pelleted samples using the DNeasy Blood & Tissue kit (Qiagen, Valencia, California, USA). All the steps for sequencing library preparation followed exactly the protocol used by Basta and colleagues [89], including (i) DNA shearing by sonication (to produce 200- to 500-bp fragments); (ii) end repair; (iii) addition of poly(C) tail; and (iv) enrichment of transposon–genome junctions and addition of adapter for Illumina sequencing by PCR [89,90]. The resulting amplified DNA samples were sequenced using 100-bp single-end reads on the Illumina HiSeq 2500 platform at the Millard and Muriel Jacobs Genetics and Genomics Laboratory at Caltech. Data analysis also followed Basta and colleagues [89]. In summary, sequences were mapped to the *P*. *aeruginosa* UCBPP-PA14 genome sequence using Bowtie 2 [91] and were analyzed in MATLAB using the ARTIST Tn-seq analysis pipeline [92], with nonoverlapping windows of 100 bp across the genome [89,92]. Using the Mann–Whitney U statistical test, the total reads mapping for each gene in the “+PYO” samples were compared to the corresponding reads in the “No PYO” control for each replicate independently [89,92]. Next, the read ratio for each replicate was calculated within ARTIST for each gene and then log_2_ transformed. Finally, the *p*-values for both replicates were combined using the Fisher combined probability test as done in Basta and colleagues [89], and the average of the log_2_ ratios of the 2 replicates are also shown. For the log_2_ ratios and *p*-values for all PA14 genes, see S1 Table. For heatmaps shown in Fig 1A, the average log_2_ ratios (fitness) for the selected genes were plotted using the *geom_tile()* function from the ggplot2 package in R [93,94].

### Tn-seq datasets correlation analysis

To compare the results of this Tn-seq analysis with a previously published study [24] analyzing fitness determinants for survival during ciprofloxacin treatment in the *P*. *aeruginosa* PAO1 strain background (Fig 1E, S1 Table), the data from that study’s supplemental S1 Table were used. The normalized average ratio of reads in the treated sample compared to reads in the input sample for each gene (geometric mean of 3 replicates) was log_2_ transformed for comparison to the Tn-seq data described above. The list of genes was filtered to include only genes for which ratios were reported in both our PYO Tn-seq experiment and the ciprofloxacin Tn-seq study and for which there are clear orthologs in both strains (*n* = 4,209 genes). Orthologs were determined using the “pseudomonas.com” database [95].

### Tn-seq validation experiments

To validate the Tn-seq results (Fig 1C), experiments were performed by comparing the survival of 4 different mutants (Δ*phz*Δ*ackA*Δ*pta*, Δ*phz*Δ*lptA*, Δ*phz*Δ*mexS*, and Δ*phz*Δ*dctBD*) to the survival of the Δ*phz* strain upon exposure to PYO. The experimental design was very similar to the one used in for the Tn-seq, with minor adaptations. An overnight culture (5 mL) of each strain was grown in SMM (40 mM succinate) from LB plates. Cells were washed and resuspended at an OD_500_ of 0.1 (or 0.25 for Δ*phz*Δ*dctBD*) in the same medium to start the new cultures (5 mL), which were grown to OD_500_ approximately 0.8–1, pelleted, washed, and resuspended in the same minimal medium without succinate (no carbon source) at OD_500_ of 1. For each strain, the culture was split across 8 to 12 wells (150 μL cultures) in a 96-well plate, with 100 μM PYO added to half of the cultures. Moreover, 70 μL of mineral oil was added to the top of the wells to prevent evaporation. PI at 5 μM was also added to the cultures to monitor cell death [16]. The plate was then moved to a BioTek Synergy 4 plate reader (BioTek, Winooski, Vermont, USA) and incubated under shaking conditions at 37°C for 24 hours. After incubation, cultures were serially diluted in buffer and plated for CFUs on LB agar, and survival in the presence of PYO was compared to the no-PYO control. Plates were incubated at room temperature (RT), and CFUs were counted after 36 to 48 hours. In this study, a stereoscope was always used to count the CFUs. Survival levels were calculated for each mutant (i.e., for each replicate, the % survival for “+PYO” was calculated based on CFUs for “No PYO”). Then, the survival levels for each mutant were normalized by the survival levels of the Δ*phz* parent strain (i.e., % survival for “+PYO” for each mutant was divided by the average % survival for “+PYO” of the Δ*phz* strain); these “fitness” values were log_2_ transformed for plotting.

### PYO tolerance with efflux inhibitor

Survival assays with efflux inhibition were performed to test the importance of efflux systems in *P*. *aeruginosa* for tolerance against PYO toxicity. From a Δ*phz* overnight culture pre-grown in SMM (20 mM succinate), a new 7-mL culture was started in fresh SMM at an OD_500_ of 0.05 and was incubated for around 10 hours (enough to reach stationary phase). Cells were then pelleted, washed, and resuspended in MPB at an OD_500_ of 1 (10 mL of culture was prepared). The culture was then split into 4 different treatments: (i) no PYO, no PAβN; (ii) 100 μM PYO, no PAβN; (iii) no PYO, with PAβN (50 μg/mL); and (iv) 100 μM PYO, with PAβN. Each of the treatments were split across 12 wells containing 150 μL of culture + 70 μL of mineral oil in a 96-well plate. The plate was incubated at 37°C under shaking conditions using a BioTek Synergy 4 plate reader. Samples were serially diluted in MPB and plated for CFUs on LB agar after 12, 24, and 48 hours. Survival for treatments containing PYO was calculated based on the CFUs counted for the negative control without PYO (Fig 1D). At each time point, 4 wells were sampled, with each well considered an independent replicate. The experiment was repeated twice with similar results.

### Antibiotic tolerance experiments using *P*. *aeruginosa*

#### Tolerance assay for WT, Δ*phz*, and Δ*phz* + PYO

For most antibiotic tolerance assays (except for tolerance using cells harvested during log phase, see below), the experimental design shown in S4A Fig was followed. WT and Δ*phz* cells were grown from a plate into overnight cultures in GMM with 20 mM glucose. Next, WT and Δ*phz* cells were pelleted, washed, and resuspended at an OD_500_ of 0.05 in 4 independent new cultures (replicates) in GMM (10 mM glucose) per treatment. Three treatments were prepared: WT, Δ*phz* (no PYO), and Δ*phz* + 100 μM PYO, with 4 independent biological replicates for each. Each of the 4 individual cultures (replicates) were incubated for around 20 hours, reaching stationary phase, in 7-mL cultures (18 × 150 mm glass tubes). Each individual culture (replicate) was then split into a negative control (no antibiotic) or antibiotic treatment (2 mL of culture per each treatment, using plastic Falcon tubes, VWR, Cat. No. 352059, Radnor, Pennsylvania, USA). After addition of the antibiotic from concentrated stocks, cultures were incubated for 4 hours, serially diluted in MPB and then plated for CFUs on LB agar. Unless stated otherwise, cells were not washed before plating. We observed that washing the cells did not make any difference in the outcome of the experiments. In addition, washing was not feasible for the tolerance assays using smaller volumes (i.e., in 96-well plates). The only 2 experiments where washing was performed are described below (“Tolerance assay with PAβN” and “Tolerance assay to measure the lag in CFUs appearance”). In these cases, cells were washed because (i) the ciprofloxacin concentrations were higher (10 μg/mL) and more likely to affect *P*. *aeruginosa* cells on the plate; and (ii) for the case of the PAβN experiment, we wanted to avoid having cells be in contact with the inhibitor while growing on the plate. Antibiotics were used at the concentrations mentioned in figure legends. Plates were incubated at RT, and CFUs were counted after 36 to 48 hours. Plates were always checked again after 7 days to count any late-arising CFUs. Importantly, for all tolerance experiments performed in this study (including this and all experiments described below), each experiment was repeated at least twice on different days, with similar results.

The same protocol was followed for the experiment testing different concentrations of PYO (Fig 2H) and for the experiment testing how PYO impacts tolerance of different *P*. *aeruginosa* mutants with partial resistance to ciprofloxacin (CipR-21, 25, 33, and 40; S7E Fig). For the experiment testing tolerance after exposure to different phenazines (Fig 2E), all the phenazines were dissolved in a common solvent (DMSO), which was used as the negative control; these experiments were performed in a Δphz* mutant lacking not only the *phzA-G1* and *phzA-G2* operons but also all phenazine modification genes, to prevent the transformation of PCA into the other phenazines (see S5 Table). For experiments performed in SCFM (Fig 2D), the same experimental design was followed, with the exception that SCFM was used instead of GMM.

#### Tolerance assay for strains with arabinose-inducible constructs

For these experiments (Fig 2I, S6B Fig), the 20-hour cultures of each strain (Δ*phz* P_ara_:*mexGHI-opmD*, Δ*phz* P_ara_:*ahpB*, and Δ*phz* P_ara_:*katB*) were grown with and without 20 mM arabinose for induction of the controlled systems and then exposed to ciprofloxacin the same way described above. To rule out any nonspecific interference of the inducer, negative controls with and without 20 mM arabinose using the parent Δ*phz* strain (without the insertions in the *att*Tn7 site) were also done. Adding arabinose to the Δ*phz* strain did not impact tolerance levels (S6 Fig).

#### Tolerance assay with PAβN

Experiments using the efflux inhibitor PAβN (S4G Fig) were also performed similarly to the way as described above. The only differences were that after the 20 hours incubation and before the addition of the antibiotic, PAβN was added to the cultures at a final concentration of 50 μg/mL. Cultures were incubated for 15 minutes and then ciprofloxacin was added, followed by a 4-hour incubation. For these experiments, instead of plating cells directly on LB, 1 mL of culture of each replicate/treatment was pelleted (12,500 rpm for 2 minutes), washed in MPB for removal of ciprofloxacin and PAβN, and only then serially diluted in MPB and plated on LB for CFU counting.

#### Tolerance assay to measure the lag in CFUs appearance

This experiment (S4F Fig) followed the same general protocol described above (using 10 μg/mL of ciprofloxacin). The difference was that the reported CFUs were counted after incubation of LB plates for 2 days and 7 days, whereas otherwise only the final counts from the seventh day were reported. This was done to quantify lag in the CFUs’ growth under the studied conditions. Similar to the tolerance assays with PAβN and ciprofloxacin described above, cells were pelleted and washed before plating on LB for CFU counting.

#### Tolerance assay for cells harvested during log phase

Δ*phz* cells were grown in overnight cultures in GMM (20 mM glucose). Next, cells were pelleted, washed, and resuspended into 2 new cultures, one with PYO (100 μM) and one without PYO, at an OD_500_ of 0.05 in GMM (10 mM glucose, 7-mL cultures). Cultures were grown until OD_500_ = 0.5 (around 5 to 6 hours). Cells were then washed and resuspended in the same medium at an OD_500_ of 0.5, but without the nitrogen source (i.e., no NH_4_Cl). PYO was re-added after washes to the culture that was pre-grown with PYO. The cultures, one with and one without PYO, were then split into different treatments: negative control (no antibiotic), ciprofloxacin (0.5 μg/mL), levofloxacin (1 μg/mL), gentamicin (16 μg/mL), and tobramycin (4 μg/mL). Then, they were all transferred to wells in a 96-well plate (3 to 4 wells per treatment, with each well being considered an independent replicate). Cultures within wells contained 150 μL with an additional 70 μL of mineral oil on top to prevent evaporation. The depletion of nitrogen prevented growth in the negative control, which limited overestimation of the antibiotic killing effect (because survival rates were calculated relative to the negative control). The plates were incubated for 4 hours at 37°C under shaking conditions (175 rpm) using a benchtop incubator (VWR incubator orbital shaker). The 96-well plate was kept inside an airtight plastic container with several wet paper towels to maintain high humidity attached to the shaker. After incubation, cells were serially diluted and plated on LB agar for CFU counting (S4B Fig). A similar experiment was also performed with the strains containing arabinose-inducible constructs (Δ*phz* P_ara_:*mexGHI-opmD*, Δ*phz* P_ara_:*ahpB*, and Δ*phz* P_ara_:*katB*) and the Δ*phz* background control (S6A Fig), for which tolerance to ciprofloxacin (0.5 μg/mL) was tested. The experiment followed the same protocol described above, with the difference that, instead of presence or absence of PYO, strains were incubated in the presence or absence of 20 mM arabinose.

### Time-lapse microscopy experiment and quantification

Fluorescently tagged strains of WT or Δ*phz* were grown in GMM, and tolerance experiments were performed as shown in S4D Fig using ciprofloxacin (10 μg/mL). After the 4-hour incubation with the antibiotic, cells were washed and resuspended in GMM. The 2 different strains were then mixed and placed on an agarose pad containing GMM (no ciprofloxacin or PYO was added to the pad). Agarose pads were placed into a PELCO Clear Wall Glass Bottom Dish (Cat. No. 14023–20), and the dish was used for imaging within the microscope incubation chamber. Outgrowth was visualized using a Nikon Ti2E microscope with Perfect Focus System 4 (Nikon Instruments, Melville, New York, USA). Incubation proceeded for 12.5 to 15 hours at 37°C, with imaging every 15 minutes in bright field (phase contrast) and green and red fluorescence channels (50-ms exposure with 470-nm LED lamp and a green-FITC filter [ex = 465 to 495 nm, em = 515 to 555 nm] for GFP; 50-ms exposure with 555-nm LED lamp and a quad band filter [red ex = 543 to 566 nm, red em = 580 to 611 nm] for mApple).

For image analysis, a Fiji macro was used. Briefly, fluorescent channels (GFP/mApple) of the first and last time points were segmented using the “Auto Threshold” function and “Default” setting. The area of the segmented cells was then recorded using the “Analyze Particles” function in Fiji [96]. This allowed for quantification of the total area covered by cells within each channel, with each field of view being processed separately. After that, for each field of view, the total area covered by WT cells (or Δ*phz* + 100 μM PYO, depending on the experiment) was divided by the area covered by Δ*phz* cells to obtain the relative “growth area ratios”. This was done for first and last time points. Three experiments were performed, with different fluorescent protein/strain combinations: (i) WT::mApple/Δ*phz*::GFP (*n* = 13 fields of view, Fig 2F and 2G, S1 Movie); (ii) Δ*phz*::GFP+PYO /Δ*phz*::mApple (*n* = 19, Fig 2G); and (iii) WT::GFP/Δ*phz*::mApple (*n* = 16, S4E Fig). GFP/mApple were controlled by the *rpsG* promoter for all of the strains (S5 Table).

### RNA extraction and quantitative reverse transcriptase PCR (qRT-PCR)

#### Experiment 1—Measurement of PYO-induced gene expression

Six different treatments were prepared for this qRT-PCR experiment: (i) WT PA14; (ii) Δ*phz*; (iii) Δ*phz* + 1 μM PYO; (iv) Δ*phz* + 10 μM PYO; (v) Δ*phz* + 100 μM PYO; and (vi) Δ*phz* + 200 μM PYO. Cultures of WT or Δ*phz* were grown overnight in GMM (20 mM glucose), then cells were washed and resuspended at an OD_500_ of 0.05 (3 replicates) in fresh GMM (5 mL in culture tubes). Different concentrations of PYO were added to Δ*phz* cultures as mentioned, and all cultures were incubated for around 8.5 hours (until early stationary phase). This was enough time for WT to make PYO (around 50 to 70 μM, measured by absorbance at OD_691_ [97]). After incubation, cells were pelleted, immediately frozen using liquid nitrogen, and stored at −80°C.

#### Experiment 2—Measuring arabinose induction of *mexGHI-opmD*, *ahpB*, and *katB*

Eight different treatments were prepared for this qRT-PCR experiment, in which each of the 4 tested strains (Δ*phz*, Δ*phz* P_ara_:*mexGHI-opmD*, Δ*phz* P_ara_:*ahpB*, and Δ*phz* P_ara_:*katB*) were incubated with and without 20 mM arabinose for artificial induction of the constructs. Cultures of the 4 strains were grown overnight in GMM (20 mM glucose), then cells were washed and resuspended at an OD_500_ of 0.05 (3 replicates) in the same medium (5 mL in culture tubes), with and without 20 mM arabinose (for conditions without arabinose, the respective amount of water was added). Cultures were incubated for around 8.5 hours, then pelleted, immediately frozen using liquid nitrogen, and stored at −80°C.

For RNA extraction, previously published protocols were followed [16,85]. Briefly, samples were thawed on ice for 10 minutes and resuspended in 215 μL of TE buffer (30 mM Tris.Cl, 1 mM EDTA, pH 8.0) containing 15 mg/mL of lysozyme + 15 μL of proteinase K solution (20 mg/mL, Qiagen) and then incubated for 8 to 10 minutes. For lysis steps and RNA extraction, the RNeasy kit (Qiagen) was used. Samples were then treated with TURBO DNA-free kit (Invitrogen, Waltham, Massachusetts, USA) for removal of any contaminant gDNA. Next, cDNA was synthesized using iScript cDNA Synthesis kit (Bio-Rad, Hercules, California, USA) (1 μg of total RNA was used). For these kits, the manufacturer’s instructions were followed. qRT-PCR reactions were performed using iTaq Universal SYBR Green Supermix (Bio-Rad) in 20 μL reactions using a 7500 Fast Real-Time PCR System machine (Applied Biosystems, Waltham, Massachusetts, USA) following published protocols [16]. Standard curves for each primer pair were prepared using *P*. *aeruginosa* gDNA and were used for calculation of cDNA for each gene studied. The housekeeping gene *oprI* was used as a control gene for normalizations [85].

Data showing total *oprI*-normalized cDNA levels (i.e., cDNA measured for a certain gene in a certain sample, divided by the respective cDNA measured for *oprI* in the same sample) and the log_2_ fold change in expression are shown in Fig 2B and S1–S3 and S5 Figs. Fold changes were calculated relative to the mean value for Δ*phz* samples without added PYO (Fig 2B, S1B and S3 Figs) or the mean value of samples from the same strain without added arabinose (S5B Fig). cDNA values for replicates within each efflux gene/treatment (shown in S2 Fig) were averaged and used with the *geom_tile()* function in R [93,94] for generation of the heatmap shown in Fig 2B.

### *Stenotrophomonas* and *Burkholderia* growth curves and antibiotic tolerance assays

*S*. *maltophilia* ATCC 13637, *B*. *cepacia* ATCC 25416, *B*. *cenocepacia* AU42085, *B*. *multivorans* AU42096 (*B*. *multivorans 1)*, and *B*. *gladioli* AU42104 were used in the growth experiments shown in Fig 5A (for strain details, see S5 Table). Each strain was grown overnight in GMM (20 mM glucose, 5 mL culture tubes) supplemented with 1× MEM AA (MilliporeSigma, Cat. No. M5550, Burlington, Massachusetts, USA). Cells were pelleted, washed, and resuspended in new cultures at an OD_500_ of 0.05 in the same medium. Cultures were then split, different concentrations of PYO were added (0, 10, 50, or 100 μM for *S*. *maltophilia*; 0, 10, or 100 μM for all others), and moved to a 96-well plate (4 to 6 wells per treatment, with each well being considered an independent replicate). Cultures within wells contained 150 μL with an additional 70 μL of mineral oil on top to prevent evaporation. The plates were incubated at 37°C under shaking conditions using a BioTek Synergy 4 plate reader with OD_500_ measurements every 15 minutes for 24 hours to measure growth. Assays for tolerance to ciprofloxacin with or without exogenous PYO were performed for *S*. *maltophilia* (sensitive to PYO) and for 4 *Burkholderia* strains (all resistant to PYO): *B*. *cepacia*, *B*. *cenocepacia*, *B*. *multivorans 1*, and *B*. *multivorans* AU18358 (*B*. *multivorans 4*). The experiments followed exactly what was done for *P*. *aeruginosa* (S4A Fig), except that cultures were grown in GMM + AA, and are shown in Fig 4B–4E. Finally, a tolerance assay in SCFM with and without PYO was performed for *B*. *multivorans 1* (Fig 5H) and followed what was described for the SCFM experiments in *P*. *aeruginosa* (with the only difference being the ciprofloxacin concentrations, always mentioned in the legends).

### Coculture antibiotic tolerance experiments

To test how PYO produced by *P*. *aeruginosa* impacts tolerance to ciprofloxacin in other species, coculture experiments were performed using membrane-separated 12-well tissue plate cultures containing 0.1 μm pore PET membranes (VWR Cat. No. 10769–226). Briefly, overnight cultures of the *P*. *aeruginosa* strain (WT/Δ*phz* PA14 or PA 76–11) and the respective other species tested (*S*. *maltophilia*, *B*. *cepacia*, *B*. *cenocepacia*, or *B*. *multivorans 1*) were prepared in GMM (20 mM glucose) + AA. Cells were pelleted, washed, and resuspended to different ODs as follows: (i) for any *P*. *aeruginosa*–*Burkholderia* assay, *P*. *aeruginosa* starting OD_500_ = 0.05 and *Burkholderia* starting OD_500_ = 0.025; and (ii) for the *P*. *aeruginosa*–*S*. *maltophilia* assay, *P*. *aeruginosa* starting OD_500_ = 0.01 and *S*. *maltophilia* starting OD_500_ = 0.1. *P*. *aeruginosa* was cultured in the bottom part of the well (600 μL), while the other species was cultured in the upper part of the well (100 μL), as shown in Fig 5C. *B*. *cepacia* and *S*. *maltophilia* were cultured either with WT or Δ*phz P*. *aeruginosa* PA14 (with and without 100 μM PYO exogenously added).

*B*. *cenocepacia* and *B*. *multivorans 1* were cultured either with PA 76–11 (a *P*. *aeruginosa* strain isolated from CF sputum that produced 50 to 100 μM PYO in these assays) or alone in the presence or absence of 100 μM PYO. For cases where *Burkholderia* was grown alone, the strain tested was grown in both the bottom and top parts of the membrane-separated wells. In all experiments, cocultures were grown for around 20 hours at 37°C under shaking conditions (175 rpm) using a benchtop incubator, followed by addition of ciprofloxacin (concentrations were either 1 or 10 μg/mL, as specified in the figure legends) and incubation for 4 hours. The membrane-separated plates were kept inside an airtight plastic container with several wet paper towels to maintain high humidity attached to the shaker. For every coculture combination in the membrane-separated plate, 3 wells were used as a negative control (no antibiotic), and 3 wells were used for ciprofloxacin treatment; each well was considered an independent replicate. After incubation with ciprofloxacin, cells were serially diluted in MPB and plated for CFUs on LB. In most cases, only *Burkholderia* cells were plated (Fig 5F). However, to test if our *P*. *aeruginosa* WT strain was still more tolerant than the Δ*phz* strain when both were grown in the presence of a *Burkholderia* species, we performed an experiment with *P*. *aeruginosa* PA14 and *B*. *multivorans 1* where we treated the cocultures with ciprofloxacin 1 μg/mL and plated *P*. *aeruginosa* (Fig 5G). Finally, we also performed a coculture experiment in SCFM (*P*. *aeruginosa* PA14 WT/Δ*phz* with *B*. *multivorans 1*) to test if PYO produced by PA14 WT increases tolerance in *Burkholderia* in this medium (Fig 5I). This experiment in SCFM followed the same overall experimental design used before, except for using SCFM instead of GMM in all steps.

### Determination of minimum inhibitory concentrations

The antibiotic concentrations used for selecting de novo antibiotic-resistant mutants in the fluctuation tests were chosen based on the results of a modified agar dilution MIC assay. Overnight cultures were grown for each strain in GMM (with 10 mM glucose) or GMM (10 mM glucose) + AA, respectively, then diluted to an OD_500_ of 0.5, from which 3 μL was spotted onto MH agar containing a 2-fold dilution series of the antibiotic. After the spots dried, the antibiotic plates were incubated upside down for 48 hours at 37°C before assessing the spots for growth. We considered the MIC to be the first concentration at which there was neither a lawn of background growth nor dozens of overlapping colonies visible without magnification. We generally used 2 times this MIC as the selection condition for fluctuation tests; for *P*. *aeruginosa*, this corresponded to the EUCAST [27] resistance breakpoints for ciprofloxacin and levofloxacin, while our chosen concentrations of gentamicin and tobramycin were 2-fold higher than the EUCAST breakpoints [32]. EUCAST breakpoints are not available for *Stenotrophomonas* spp. or the *B*. *cepacia* complex. The appropriateness of the selection condition was additionally verified by performing a fluctuation test, as described below, and choosing the antibiotic concentration that reliably yielded a countable number of colonies (0 to several dozen, with at least several nonzero counts per 44 parallel cultures) in each well.

Ciprofloxacin MICs for parent strains and isolated mutants from the fluctuation tests were determined according to standard clinical methods for broth microdilution assays [27]. In brief, cells from either overnight cultures in MH broth or fresh streaks on LB agar (14 to 16 hours old) were resuspended to a density of 3 to 7 × 10^5^ CFUs/mL in a 2-fold dilution series of ciprofloxacin in MH broth, with or without 100 μM PYO. The dilution series were set up in a final volume of 100 μL per well in 96-well microtiter plates, with appropriate no-antibiotic and cell-free controls. Three biological replicates (independent overnight cultures or cell suspensions) were prepared for each tested strain. Following inoculation, the microtiter plates were sealed with a plastic film to prevent evaporation and incubated in a single layer at 37°C, without shaking. The wells were assessed for growth (turbidity) visible to the naked eye after 18 hours of incubation.

### Fluctuation tests, calculation of mutation rates, and model fitting

For all tested strains and conditions, fluctuation tests were performed by inoculating 200-μL cultures in parallel in a flat-bottomed 96-well plate. All reported fluctuation test data for *P*. *aeruginosa* are from experiments using the Δ*phz* strain. We also performed fluctuation tests using the *P*. *aeruginosa* PA14 WT strain and performed phenotypic and genotypic characterization of partially resistant mutants detected in those experiments (see below); however, the effect of PYO on apparent mutation rates in WT was difficult to interpret due to inconsistent PYO production in the 96-well plates. For cultures that were grown with PYO (or arabinose in the case of strains with arabinose-inducible constructs), the PYO (or 20 mM arabinose) was added to the medium before inoculation. The cultures were inoculated with a 10^−6^ dilution of a single overnight culture (representing a biological replicate) that had first been diluted to a standard OD_500_ of 1.0, corresponding to an initial cell density of approximately 2,000 to 2,500 CFUs/mL (400 to 500 cells/culture). Each treatment condition consisted of 44 such parallel cultures.

The 96-well plates were placed inside an airtight plastic container with several wet paper towels to maintain high humidity, then incubated at 37°C with shaking at 250 rpm. For plating during log phase, the cultures were incubated until reaching approximately half-maximal density (OD_500_ of 0.4 to 0.7 for *P*. *aeruginosa* in GMM with 10 mM glucose or 0.9 to 1.2 for *P*. *aeruginosa* in SCFM or *B*. *multivorans* in GMM + AA). For plating during stationary phase, the cultures were incubated for 24 hours. The cultures were then plated by spotting 40 to 50 μL per culture into single wells of 24-well plates (for any given experiment, the same volume was spotted for all parallel cultures); each well contained 1 mL of MH agar or SFCM agar plus an antibiotic, with or without 100 μM PYO (or 20 mM arabinose for strains with arabinose-inducible constructs). In the case of *B*. *multivorans* cultures that were spotted onto antibiotic plates containing 100 μM PYO, the cultures were first diluted 1:10 (if not pretreated with 100 μM PYO) or 1:100 (if pretreated with 100 μM PYO).

At the same time as plating onto the antibiotic plates, 6 representative cultures from each treatment were serially diluted and plated on LB agar plates to assess total CFUs. The antibiotic plates were incubated upside down, in stacks of no more than 8, at 37°C for 16 to 24 hours for *P*. *aeruginosa* (except for gentamicin plates, which were incubated for 40 to 48 hours) or 40 to 48 hours for *B*. *multivorans*. Subsequently, colonies were counted under a stereoscope at the highest magnification for which the field of view still encompassed an entire well; occasionally, a well contained too many colonies to count (a so-called “jackpot” culture [98]), in which case that culture was discarded from further analysis. The LB agar plates for total CFU counts were incubated for 30 to 36 hours at RT before counting colonies at the same magnification.

Mutation rates reported in the figures were calculated using the function newton.LD.plating from the R package rSalvador [99] to estimate *m*, the expected number of mutations per culture. This is a maximum likelihood-based method for inferring mutation rates from fluctuation test colony counts, based on the classic LD distribution with a correction to account for the effects of partial plating (i.e., plating a portion of each culture rather than the total volume) [99]. We chose this method because it has been shown to be the most accurate estimator of *m* when partial plating is involved [99,100]. To get μ_app_ (apparent mutation rate per generation) from *m*, we divided *m* by the total number of cells per parallel culture [99], as estimated from the mean number of CFUs counted for the 6 representative cultures.

To compare the fits of different formulations of the LD distribution to our data, we generated theoretical cumulative distributions using the parameter values estimated for our data. Specifically, for the Hamon and Ycart version of the LD model [44], we estimated *m* and *w* (relative fitness of mutants compared to the parent strain in the nonselective pre-plating liquid growth medium) using the function GF.est from the R script available at http://ljk.imag.fr/membres/Bernard.Ycart/LD/ (version 1.0; note that in the script, *m* is called alpha and 1/*w* is called rho); then, we used the function pLD from the same script to generate the theoretical distribution. For the mixed LD–Poisson and basic LD models, we wrote and used an R translation of the MATLAB code written by Lang and colleagues [45]; the original code is available at https://github.com/AWMurrayLab/FluctuationTest_GregLang. The basic LD model used by Lang and colleagues [45] is equivalent to that available in the rSalvador package (using the function newton.LD), except without the correction for partial plating; the latter is only important when using the estimate of *m* to infer the mutation rate, not when comparing the fits of different models to the empirical cumulative distribution of the raw colony counts.

Plots of the empirical cumulative distributions of our data against the theoretical models showed that the Hamon and Ycart model was a visually good fit in all cases (see Figs 4B and 6C and S9 Fig for examples). To further assess goodness of fit of the Hamon and Ycart model, we performed Pearson chi-squared test in R after binning the data and theoretical distribution such that the expected number of cultures in each bin of mutant counts was at least 5 [101]. To compare the goodness of fit of the Hamon and Ycart model to the basic LD model, we calculated the negative log likelihood for each model and performed the likelihood ratio test. To compare the Hamon and Ycart model to the mixed LD–Poisson model, we simply compared the negative log likelihoods (smaller values indicate a better fit); the likelihood ratio test was not applicable as these 2 models contain the same number of parameters. Note that although the Hamon and Ycart (or in some cases, LD–Poisson) models were often better fits than the basic LD model, we still used the basic LD model for statistical comparison of mutation rates between conditions, because an accurate method to account for partial plating has not yet been developed for the cases of post-plating mutations or differential fitness between mutants and parent strains [99]. Nevertheless, similar patterns in mutation rates were observed when using an older method of accounting for partial plating to derive μ_app_ from the Hamon and Ycart model [102]; the Pearson correlation coefficient was 0.98 for mutation rates calculated with the newton.LD.plating function in rSalvador versus the partial plating–corrected Hamon and Ycart method (S3 Table). We also separately performed nonparametric statistical analysis of the raw mutant frequencies (i.e., mutant colony counts divided by the number of cells per parallel culture), as such analysis is agnostic to any assumptions about the biological processes underlying the data. The statistical significance of this analysis generally corresponded with the statistical significance of a likelihood ratio test based on the newton.LD.plating model of mutation rates, indicating that the effects of PYO were robust to different mathematical approaches to analyzing the fluctuation test data (S2 Table).

### Characterization of antibiotic resistance phenotypes

We defined putative ciprofloxacin-resistant mutants as “enriched” by PYO in the fluctuation tests if colonies with a given morphology were at least 2 times more numerous on the PYO-containing ciprofloxacin plate than the respective non-PYO-containing ciprofloxacin plate derived from the same 200-μL culture. These putative mutants could be either from the PYO pretreated or non-PYO pretreated branches of the fluctuation test. Putative mutants that were seemingly enriched by PYO were restreaked for purity on PYO-containing agar plates at the same ciprofloxacin concentration on which they were selected in the fluctuation test (0.5 μg/mL for *PA* and 8 μg/mL for *B*. *multivorans*). Putative mutants that were not enriched by PYO were restreaked on ciprofloxacin agar plates without PYO. Frozen stocks of each restreaked, visually pure isolate were prepared by inoculating cultures with single colonies in 5 mL of liquid LB, incubating to stationary phase, mixing 1:1 with 50% glycerol, and storing at −80°C.

The levels of ciprofloxacin resistance of selected isolates, as well as the parent strains, were assessed using a CFU recovery assay as follows. For each isolate, four 5 mL cultures in GMM (for *P*. *aeruginosa*) or GMM + AA (for *B*. *multivorans*) were inoculated directly from the frozen stock to minimize the number of generations in which secondary mutations could be acquired. The cultures were grown to stationary phase overnight, then subcultured to an OD_500_ of 0.05 in 5 mL of fresh GMM (for *P*. *aeruginosa*) or GMM + AA (for *B*. *multivorans*), with or without 100 μM PYO. The new cultures were grown to mid-log phase, then serially diluted in GMM or GMM + AA (+/− 100 μM PYO as appropriate) and plated for CFUs (10 μL per dilution step) on (1) plain MH agar; (2) MH agar + ciprofloxacin; and (3) MH agar + ciprofloxacin + 100 μM PYO. The lowest plated dilution was 10^−1^, making the limit of detection approximately 1,000 CFUs/mL.

### Identification of mutations by whole-genome sequencing

gDNA was isolated from selected putative mutants and the parent strains using the DNeasy Blood & Tissue kit (Qiagen). Library preparation and 2 × 150 bp paired-end Illumina sequencing was performed by the Microbial Genome Sequencing Center (Pittsburgh, Pennsylvania, USA), with a minimum of 300-Mb sequencing output per sample (approximately 50 × coverage). Forward and reverse sequencing reads were concatenated into a single file for each isolate, and quality control was performed using Trimmomatic (version 0.39) [103] with the following settings: LEADING:27 TRAILING:27 SLIDINGWINDOW:4:27 MINLEN:35. Mutations were then identified using breseq (version 0.34.1) [104]. The annotated reference genome for *P*. *aeruginosa* UCBPP-PA14 was obtained from BioProject accession number PRJNA38507. For *B*. *multivorans* AU42096, no reference genome was available from NCBI. Therefore, a genome scaffold was assembled from the paired-end sequencing data for the parent strain using SPAdes (version 3.14.0) with default parameters [105]. This scaffold was then used as the reference for breseq. Differences between the parent strain and isolates were identified using the gdtools utility that comes with breseq to compare the respective breseq outputs. All sequenced *P*. *aeruginosa* mutants were derived from the Δ*phz* strain except for CipR-33 and CipR-40, which were derived from the WT strain. In the case of *B*. *multivorans*, several dozen putative mutations were identified that were common to all 3 sequenced putative mutants. We assumed that these represented assembly errors in the parent strain genome scaffold, but even if they were genuine mutations, these would not account for the phenotypic differences between the isolates; therefore, S6 Table reports only mutations that were unique to each isolate. The genomic loci containing each putative mutation for the *B*. *multivorans* isolates were identified by retrieving the surrounding 200 bp from the parent genome scaffold and using the nucleotide BLAST tool on the MicroScope platform [106] to find the closest match in the *B*. *multivorans* ATCC 17616 genome.

### Growth curves with propidium iodide

To verify that PYO did not increase the population turnover rate (i.e., cell death) in our log-phase fluctuation tests prior to the antibiotic selection step, we performed growth curves in the presence of different concentrations of PYO, with the addition of PI as a fluorescent marker for cell death. The use of PI as a marker for PYO-induced cell death has previously been validated under similar conditions [16]. The growth curves were performed in GMM and SCFM for *P*. *aeruginosa* Δ*phz* and GMM + AA for *B*. *multivorans 1*. The cultures were prepared and incubated in the same manner as the fluctuation tests, except that 5 μM PI was added at the beginning of the experiment, and measurements for OD_500_ and PI fluorescence (ex = 535 nm, em = 617 nm) were taken periodically using a Spark 10M plate reader (Tecan US, Morrisville, North Carolina, USA). Importantly, PI stock concentration (5 mM, 1,000×) was prepared in DMSO, and the final concentration of DMSO in the cultures did not exceed 0.1%. In addition, black 96-well plates with clear bottoms were used to minimize the effects of adjacent wells on fluorescence readings.

### Statistical analyses

All statistical analyses were performed using R [94]. Welch unpaired *t* tests or 1-way analysis of variance (ANOVA) with post hoc Tukey honestly significant difference (HSD) test for multiple comparisons were used for tolerance assay data. The likelihood ratio test as implemented by the rSalvador function LRT.LD.plating was used to compare mutation rates, alongside the alternative criterion of nonoverlapping 84% confidence intervals as a proxy for the *p* < 0.05 threshold for statistical significance. The Mann–Whitney U test was used to compare the distributions of mutant frequencies. Welch unpaired *t* tests were used for comparisons of CFU recovery on ciprofloxacin plates under different PYO treatments. Benjamini–Hochberg corrections were used in all cases to control false discovery rates, except where Tukey HSD test was performed. For all antibiotic tolerance assays measured by CFUs, survival data were log_10_ transformed before statistical analyses.

## Supporting information

S1 Fig Effects of different concentrations of PYO on the expression of the *P*. *aeruginosa* oxidative stress response genes *ahpB* and *katB*. (**A)** Normalized cDNA levels measured by qRT-PCR. cDNA measurements were normalized by levels of the housekeeping gene *oprI* (see Methods). **(B)** Fold change in expression upon PYO treatment, relative to the measurements in untreated Δ*phz*. *ahpB*: alkyl hydroperoxide reductase B; *katB*: catalase B. Black horizontal lines mark the mean value for independent biological cultures (*n* = 3). The data underlying this figure can be found in Table G in S1 Data. PYO, pyocyanin; qRT-PCR, quantitative reverse transcriptase PCR.
(TIF)

S2 Fig Effects of PYO on the expression of *P*. *aeruginosa* RND efflux systems (normalized cDNA levels). The normalized cDNA levels for genes within operons coding for the 11 main RND efflux systems in *P*. *aeruginosa* are shown. cDNA levels for each gene were measured by qRT-PCR during early stationary phase and normalized by the levels of the housekeeping gene *oprI* (see Methods). This dataset was used to make the heatmap presented in Fig 2B. Black horizontal lines mark the mean value for independent biological cultures (*n* = 3). The data underlying this figure can be found in Table H in S1 Data. PYO, pyocyanin; qRT-PCR, quantitative reverse transcriptase PCR; RND, resistance-nodulation-division.
(TIF)

S3 Fig Effects of PYO on the expression of *P*. *aeruginosa* RND efflux systems (fold change). The PYO-induced changes in expression for genes within operons coding for the 11 main RND efflux systems in *P*. *aeruginosa* are shown. These plots are derived from the normalized cDNA dataset shown in S2 Fig. Here, the values for Δ*phz* were used as the basis for calculation of changes of expression (shown as log_2_ fold change). Black horizontal lines mark the mean value for independent biological cultures (*n* = 3). The data underlying this figure can be found in Table H in S1 Data. PYO, pyocyanin; RND, resistance-nodulation-division.
(TIF)

S4 Fig Effects of PYO on *P*. *aeruginosa* tolerance to different antibiotics. **(A)** Experimental design for survival assay to measure tolerance to clinical antibiotics. In conditions with exogenous PYO, the PYO was added when cultures were inoculated. PYO itself was not lethal under these experimental conditions (see panel C in this Fig). **(B)** Tolerance levels of Δ*phz* cells harvested in log phase, following growth in GMM in the presence or absence of PYO (100 μM), to different aminoglycosides and fluoroquinolones (GEN, TOB, CIP, and LVX). Data points represent replicates (*n* = 4 for all except tobramycin, for which *n* = 3). Stationary-phase tolerance experiments in GMM are not shown for the aminoglycosides (GEN and TOB), as treatment with TOB in stationary phase under our conditions at this clinically relevant concentration [32] did not result in cell death, regardless of the presence of PYO. However, for experiments performed with stationary-phase cells in SCFM, killing did happen (see Fig 2D). **(C)** Representative data showing CFUs counted for Δ*phz* grown for 20 hours (in GMM, see Methods) in the presence and absence of PYO in our tolerance assays, showing that PYO itself was not toxic under the studied conditions (*n* = 4). These are the CFUs for the negative control (no antibiotic) for the experiment performed with CIP in Fig 2C. **(D)** Experimental design for time-lapse microscopy experiments, in which cells were grown on agarose pads after exposure to CIP (10 μg/mL) in GMM. The strain/fluorescent protein examples shown (i.e., WT::mApple, Δ*phz*::GFP) are the ones used in the images of Fig 2F and S1 Movie. **(E)** Quantification of microscopy data as done in Fig 2G, but for the experiment with swapped fluorescent proteins. **(F)** Experiment quantifying how PYO affects lag for CFUs appearing after treatment with CIP (10 μg/mL, see Methods) in GMM. Treating *P*. *aeruginosa* cells with 10 μg/mL resulted in high killing levels (see panel G), and we observed an increased lag in the absence of PYO (this supports microscopy data presented in Figs 2F and 2G and S4E Fig). The survival levels were calculated for CFUs counted after 2 days (too early, since more CFUs appeared later, changing the calculated survival levels) and 7 days (no CFUs appeared after this time point; correct survival rate) of the LB plates incubation. Few new CFUs arose for WT and Δ*phz*+PYO treatments after 2 days, while several appeared for Δ*phz*. Numbers represent the mean survival ratio of WT/Δ*phz* and Δ*phz*+PYO/Δ*phz*. For survival calculated after 2 days, PYO’s presence gave the impression of an approximately 10-fold higher survival rate. However, this was mostly due to lag of Δ*phz*, and the real survival difference was around approximately 2- to 3-fold (calculated after 7 days) (*n* = 4). **(G)** Effects of the efflux inhibitor PAβN on tolerance levels to CIP of Δ*phz* cells grown in GMM in the presence or absence of PYO (100 μM). Cultures were treated with low (left) and high (right) CIP concentrations. Experiments for all the conditions were done in parallel (see Methods for full protocol) (*n* = 4). Statistics: B, C, E, G—Welch unpaired *t* tests. F—1-way ANOVA with Tukey HSD multiple comparison test, with asterisks showing the statistical significance of comparisons with the Δ*phz* (no PYO) (* *p* < 0.05, ** *p* < 0.01, *** *p* < 0.001, n.s. *p* > 0.05). Black horizontal lines mark the mean value for independent cultures (or fields of view, for E). The data underlying this figure can be found in Table I in S1 Data. ANOVA, analysis of variance; CFU, colony-forming unit; CIP, ciprofloxacin; GEN, gentamicin; GMM, glucose minimal medium; HSD, honestly significant difference; LVX, levofloxacin; PAβN, phenylalanine-arginine β-naphthylamide; PYO, pyocyanin; TOB, tobramycin; WT, wild-type.
(TIF)

S5 Fig Artificial induction of *mexGHI-opmD*, *ahpB*, and *katB*. **(A)** Normalized cDNA levels measured by qRT-PCR. cDNA levels were normalized by the housekeeping gene *oprI*. **(B)** Fold change in expression upon arabinose induction. This dataset can be compared to the PYO-mediated induction of the same genes as shown in Fig 2B and S1–S3 Figs. The 4 strains shown are (1) the parent Δ*phz* (white background); (2) Δ*phz* P_ara_:*mexGHI-opmD* (magenta background); (3) Δ*phz* P_ara_:*ahpB* (green background); and (4) Δ*phz* P_ara_:*katB* (blue background). +/− represent addition or not of 20 mM arabinose to the cultures for the artificial induction of expression. For additional experimental details and strain information, see Methods and S5 Table. Black horizontal lines mark the mean value for independent biological cultures (*n* = 3). The data underlying this figure can be found in Table J in S1 Data. PYO, pyocyanin; qRT-PCR, quantitative reverse transcriptase PCR.
(TIF)

S6 Fig Effect of artificial induction of PYO-induced genes on tolerance to CIP. Survival relative to the no-antibiotic control is shown for the parent Δ*phz* strain and the 3 arabinose-inducible strains (in which the PYO-inducible genes *mexGHI-opmD*, *ahpB*, or *katB* are under control of an arabinose-inducible promoter) grown in the presence or absence of 20 mM arabinose, without exposure to PYO. The tolerance experiments were performed for cultures in both log phase (A, *n* = 3) and stationary phase (B, *n* = 4) in GMM. In B, the experiment for *mexGHI-opmD* is the same as in Fig 2I but is also shown here for ease of comparison. Statistics: Welch unpaired *t* tests (* *p* < 0.05, ** *p* < 0.01, *** *p* < 0.001, n.s. *p* > 0.05). Black horizontal lines mark the mean value for independent cultures. The data underlying this figure can be found in Table K in S1 Data. CIP, ciprofloxacin; GMM, glucose minimal medium; PYO, pyocyanin.
(TIF)

S7 Fig Effect of PYO or PYO-induced genes on apparent mutation rates in *P*. *aeruginosa*. **(A)** Apparent mutation rates of stationary-phase Δ*phz* grown in liquid GMM and plated onto MH agar containing CIP (0.5 μg/mL), with or without pre- and/or co-exposure to 100 μM PYO relative to the antibiotic selection step (*n* = 8). **(B-C)** Apparent mutation rates of log-phase cells grown in GMM and plated onto MH agar containing CIP (0.5 μg/mL), with or without pre- and/or co-exposure to 20 mM arabinose relative to the antibiotic selection step. Data are shown for Δ*phz* P_ara_:*mexGHI*-*opmD* alone (B, left, *n* = 4) and in comparison to Δ*phz* (B, right) or Δ*phz* P_ara_:*katB* and Δ*phz* P_ara_:*ahpB* (C, *n* = 4). In all panels, each data point represents 44 parallel cultures from a single biological replicate, and the vertical lines represent 84% confidence intervals, for which lack of overlap corresponds to statistical significance at the *p* < 0.05 level [99]. The PYO treatments correspond to the following: −/− denotes no PYO pretreatment (in the liquid culture stage) or co-treatment (in the antibiotic agar plates), +/− denotes PYO pretreatment but no co-treatment, −/+ denotes PYO co-treatment without pretreatment, and +/+ denotes both PYO pretreatment and co-treatment. The data underlying this figure can be found in Table L in S1 Data. CIP, ciprofloxacin; GMM, glucose minimal medium; MH, Mueller–Hinton; PYO, pyocyanin.
(TIF)

S8 Fig Effect of PYO on resistance phenotypes and antibiotic tolerance of *P*. *aeruginosa* mutants. **(A)** The percentage of CFUs recovered on CIP (0.5 μg/mL) either with or without 100 μM PYO in the agar, for log-phase cultures of representative resistant mutants of *P*.*a*. that were not enriched by exposure to PYO in the fluctuation tests. The mutants were pre-grown with or without 100 μM PYO in GMM before plating. On the x-axis, “pre” denotes the presence of PYO in the liquid cultures and “co” denotes the presence of PYO in the agar plates. Percentage recovery was calculated relative to total CFUs counted on nonselective plates (*n* = 4). **(B)** Tolerance to CIP (1 μg/mL) of partially resistant mutants grown in GMM to stationary phase with or without 100 μM PYO (*n* = 4). Experiments were performed as shown in S5A Fig. **(C)** The percentage of CFUs recovered on CIP (0.5 μg/mL) for log-phase cultures of representative resistant mutants that were enriched by exposure to PYO in the fluctuation tests (*n* = 4). Experiments were performed in the same way as in panel A. **(D, E)** Growth curves performed for *P*. *aeruginosa* Δ*phz* (*P*.*a*.) and *B*. *multivorans 1* (*B*.*m*.) in GMM (with the addition of AA for *B*. *multivorans*; see Methods) or SCFM, with different concentrations of PYO in the presence of 5 μM PI, which is a fluorescent marker for cell death. OD_500_ (cell density) is plotted in G, while PI fluorescence is plotted in H. Gray shaded regions represent the standard deviation of 4 biological replicates. In G, the dashed horizontal lines mark the cell density at which *P*. *aeruginosa* (lower line in left panel) or *B*. *multivorans* (upper line in left panel) would have been plated in our fluctuation tests. Note that these OD_500_ values differ from those reported in the Methods section for fluctuation tests due to the use of a microtiter plate reader in this experiment, whereas a different spectrophotometer was used in the fluctuation tests. In H, the vertical dashed lines mark the time at which the cultures would have been plated in the fluctuation tests (in the left panel, left line = *B*.*m*. sampling time, right line = *P*.*a*. sampling time). The increase in fluorescence seen for *B*. *multivorans* prior to stationary phase likely reflects the production of a fluorescent metabolite rather than early cell death, as fluorescence was initially higher for the cultures not treated with PYO and the exponential phase growth rates were identical regardless of PYO treatment. Statistics: A–C—Welch unpaired *t* tests (* *p* < 0.05, ** *p* < 0.01, *** *p* < 0.001, n.s. *p* > 0.05). Unless indicated otherwise with brackets, statistical significance is shown for the comparison with the untreated (no PYO) condition. In A–C, data points represent independent biological cultures, with horizontal black lines marking the mean value for each condition. The data underlying this figure can be found in Table M in S1 Data. AA, amino acid; CFU, colony-forming unit; GMM, glucose minimal medium; *P*.*a*., *P*. *aeruginosa*; PI, propidium iodide; PYO, pyocyanin; SCFM, synthetic cystic fibrosis sputum medium.
(TIF)

S9 Fig Goodness of fit of different mathematical models for *P*. *aeruginosa* Δ*phz* fluctuation test data. Data are plotted for different combinations of PYO in liquid (pretreatment) and PYO in agar (co-exposure to antibiotic selection). The empirical CDFs of the data (black) are plotted against (1) a variation of the LD model fit with 2 parameters, *m* (the expected number of mutations per culture) and *w* (the relative fitness of mutant cells vs. WT), as implemented by Hamon and Ycart [44] (pink); (2) a mixed LD and Poisson distribution fit with 2 parameters, *m* and *d* (the number of generations that occur post-plating), allowing for the possibility of post-plating mutations, as implemented by Lang and colleagues [45] (blue); (3) the basic LD distribution model fit only with *m*, as implemented by Lang and colleagues [45] (gray). In each condition, the plotted data represent the biological replicate with the lowest chi-squared goodness of fit *p*-value (i.e., least good fit) for the Hamon and Ycart model. The data underlying this figure can be found in Table N in S1 Data. CDF, cumulative distribution function; GEN, gentamicin; LD, Luria–Delbrück; LVX, levofloxacin; PYO, pyocyanin; TOB, tobramycin; WT, wild-type.
(TIF)

S1 Table Read ratios for each gene (PA14 genome) in the PYO tolerance Tn-seq experiment. Analysis was done using ARTIST software (see Methods). Ratios = reads + PYO conditions / reads no PYO condition (log_2_ transformed), where high ratio values = increased fitness and low ratio values = decreased fitness. CIP Tn-seq values are from Cameron and colleagues [24] (see Methods). NA, not applicable. NR, not reported, meaning that there were no reads from the gene/locus within the replicate (or in at least one of the replicates, when displayed in the “average log_2_-transformed ratio” column). CIP, ciprofloxacin; PYO, pyocyanin; Tn-seq, transposon sequencing.
(XLSX)

S2 Table Statistical significance of comparisons of mutation rates and mutant frequencies. Mutation rates reported in this table were calculated using the rSalvador function newton.LD.plating and were compared using the LRT.LD.plating function to determine statistical significance. Mutant frequencies were compared using the Mann–Whitney U test. Reported *p*-values were adjusted with the Benjamini–Hochberg correction to control the false discovery rate. LD, Luria–Delbrück; LRT, likelihood ratio test.
(XLSX)

S3 Table Fluctuation test analysis results for all log-phase experiments conducted in minimal medium. Model parameters: μ, apparent mutation rate per generation; m, expected number of mutational events per cultures; w, fitness ratio of mutants/WT; d, number of post-plating generations. See Methods for details on the different mathematical models. CIP, ciprofloxacin; GEN, gentamicin; HY, Hamon and Ycart; LD, Luria–Delbrück; LRT, likelihood ratio test; LVX, levofloxacin; score, negative log likelihood; TOB, tobramycin. WT, wild-type.
(XLSX)

S4 Table Mutations detected in CIP-resistant isolates of *Pseudomonas aeruginosa* PA14. Mutations were detected using breseq, with the reference set as the *P*. *aeruginosa* UCBPP-PA14 genome obtained from BioProject accession number PRJNA38507. Pseudogenes and synonymous substitution mutations were omitted from the table. CIP, ciprofloxacin.
(XLSX)

S5 Table Strains, plasmids, and primers used in this study. This table contains a list of the strains, plasmids, and primers used in this study.
(XLSX)

S6 Table Mutations detected in partially CIP-resistant isolates of *Burkholderia multivorans* AU42096. Mutations were detected using breseq, with a draft assembly of the genome for *B*. *multivorans* AU42096 as the reference. Only mutations unique to each isolate are included. CIP, ciprofloxacin.
(XLSX)

S7 Table CIP MICs for fluctuation test isolates and parent strains. MICs were determined in a microbroth dilution assay according to standard clinical methods (see Methods). Where a range of values is presented, this indicates that the observed MIC sometimes varied depending on the initial cell density of the inoculum, even within the clinically acceptable range of 3–7 × 10^5^ CFUs/mL. CFU, colony-forming unit; CIP, ciprofloxacin; MIC, minimum inhibitory concentration.
(XLSX)

S1 Movie PYO effect on lag during outgrowth after exposure to CIP. Four time points of this movie are shown in Fig 2F. For experimental details, see Fig 2F and Methods. WT cells (PYO producers, expressing mApple) are shown in magenta, while Δ*phz* cells (PYO nonproducers, expressing GFP) are shown in green. Recovery in the absence of PYO after exposure to ciprofloxacin was imaged every 15 minutes for 15 hours. Scale bar: 20 μm. CIP, ciprofloxacin; PYO, pyocyanin; WT, wild-type.
(MP4)

S1 Data Raw data and code for reproducing all main and supporting figures. This zipped folder contains Excel tables organized by figure, within which the raw data for individual panels are displayed in separate tabs, as well as a text file containing the R code for all functions that were written especially for this manuscript.
(ZIP)

## Abbreviations

AA: amino acid
ANOVA: analysis of variance
CF: cystic fibrosis
CFU: colony-forming unit
EUCAST: European Committee on Antimicrobial Susceptibility Testing
gDNA: genomic DNA
GMM: glucose minimal medium
HSD: honestly significant difference
LB: Luria–Bertani
LD: Luria–Delbrück
MH: Mueller–Hinton
MIC: minimum inhibitory concentration
MOPS: 4-morpholinepropanesulfonic acid
MPB: minimal phosphate buffer
PAβN: phenylalanine-arginine β-naphthylamide
PCA: phenazine-1-carboxylic acid
PI: propidium iodide
PYO: pyocyanin
qRT-PCR: quantitative reverse transcriptase PCR
RND: resistance-nodulation-division
ROS: reactive oxygen species
RT: room temperature
SCFM: synthetic cystic fibrosis sputum medium
SMM: succinate minimal medium
Tn-seq: transposon sequencing
WT: wild-type
