# Supplementary figures and images for: Bacterial defenses against a natural antibiotic promote collateral resilience to clinical antibiotics
Source: PLoS Biol. 2021 Mar 10;19(3):e3001093. doi: 10.1371/journal.pbio.3001093 (PMC7946323; doi:10.1371/journal.pbio.3001093)

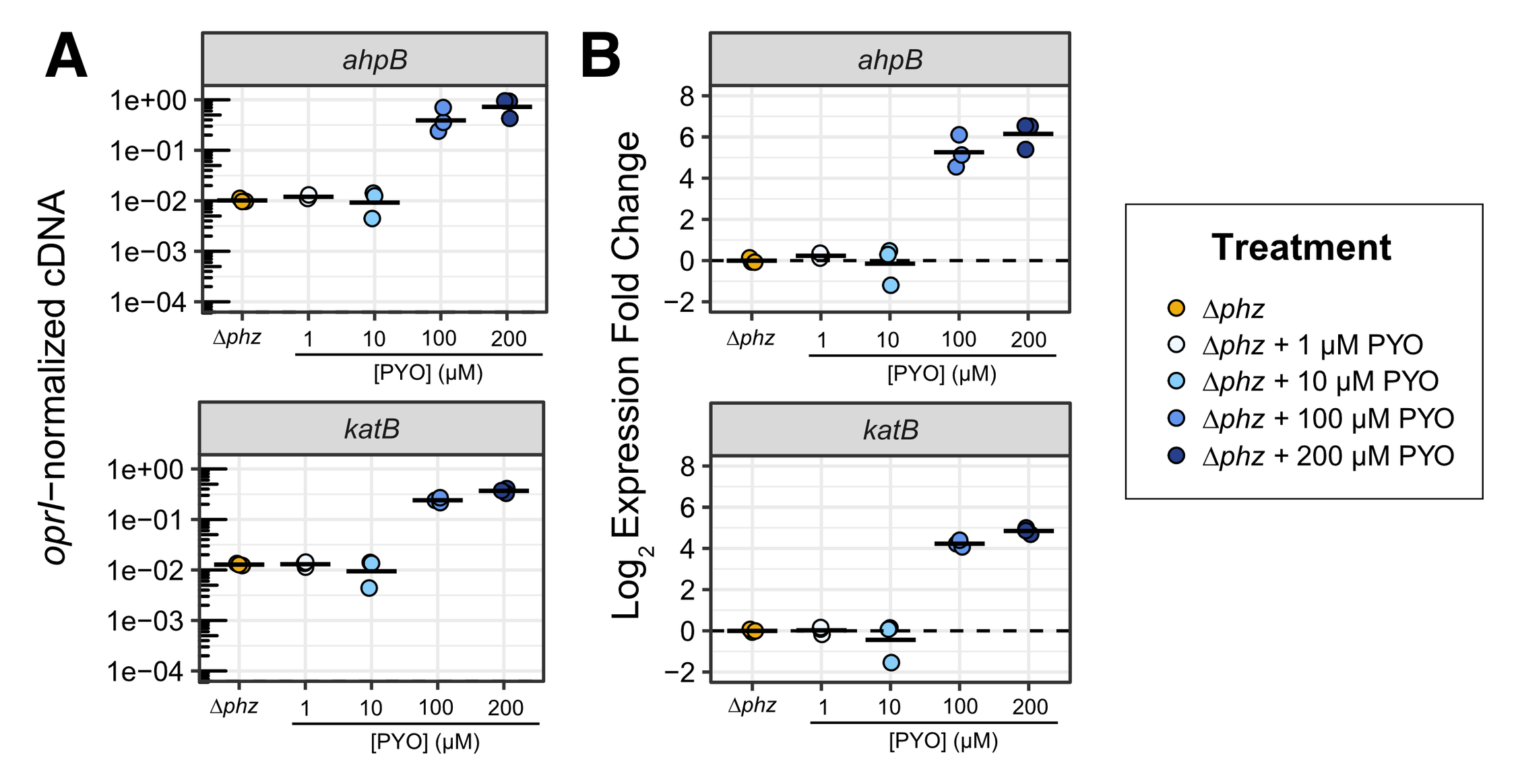

Supplement: S1 Fig — (A) Normalized cDNA levels measured by qRT-PCR. cDNA measurements were normalized by levels of the housekeeping gene oprI (see Methods). (B) Fold change in expression upon PYO treatment, relative to the measurements in untreated Δphz. ahpB: alkyl hydroperoxide reductase B; katB: catalase B. Black horizontal lines mark the mean value for independent biological cultures (n = 3). The data underlying this figure can be found in Table G in S1 Data. PYO, pyocyanin; qRT-PCR, quantitative reverse transcriptase PCR. (TIF) [file pbio.3001093.s001.tif]

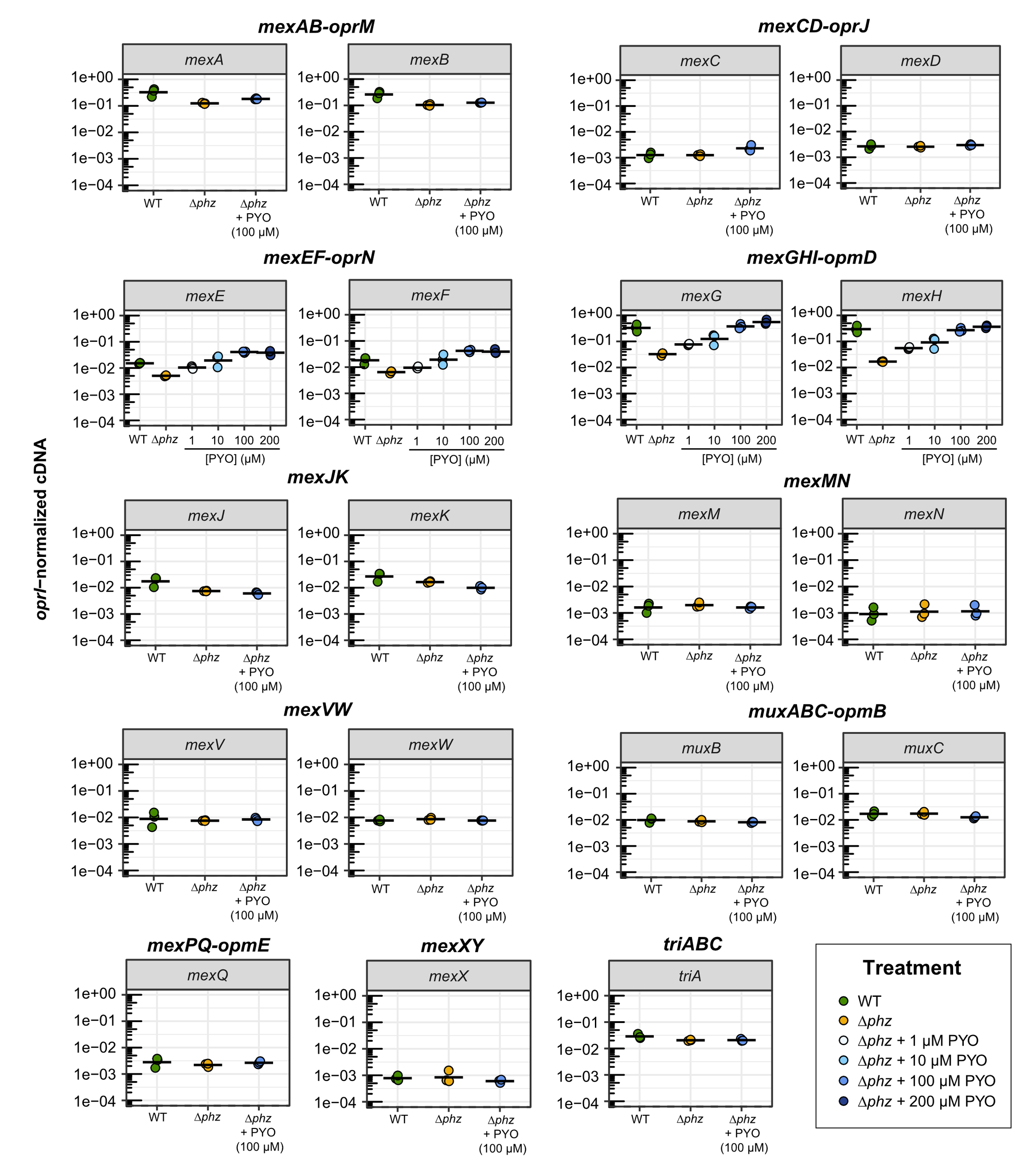

Supplement: S2 Fig — The normalized cDNA levels for genes within operons coding for the 11 main RND efflux systems in P. aeruginosa are shown. cDNA levels for each gene were measured by qRT-PCR during early stationary phase and normalized by the levels of the housekeeping gene oprI (see Methods). This dataset was used to make the heatmap presented in Fig 2B. Black horizontal lines mark the mean value for independent biological cultures (n = 3). The data underlying this figure can be found in Table H in S1 Data. PYO, pyocyanin; qRT-PCR, quantitative reverse transcriptase PCR; RND, resistance-nodulation-division. (TIF) [file pbio.3001093.s002.tif]

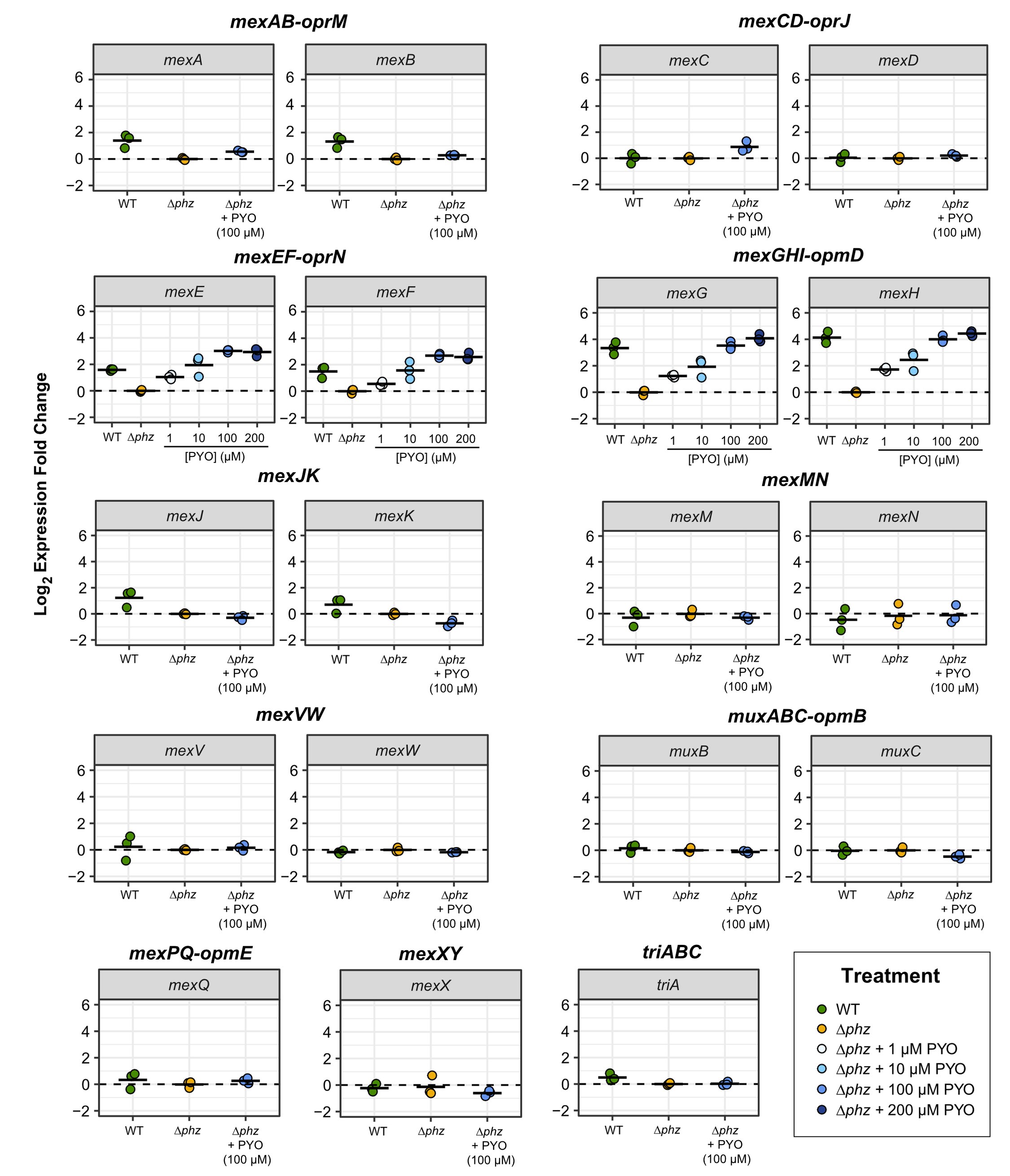

Supplement: S3 Fig — The PYO-induced changes in expression for genes within operons coding for the 11 main RND efflux systems in P. aeruginosa are shown. These plots are derived from the normalized cDNA dataset shown in S2 Fig. Here, the values for Δphz were used as the basis for calculation of changes of expression (shown as log2 fold change). Black horizontal lines mark the mean value for independent biological cultures (n = 3). The data underlying this figure can be found in Table H in S1 Data. PYO, pyocyanin; RND, resistance-nodulation-division. (TIF) [file pbio.3001093.s003.tif]

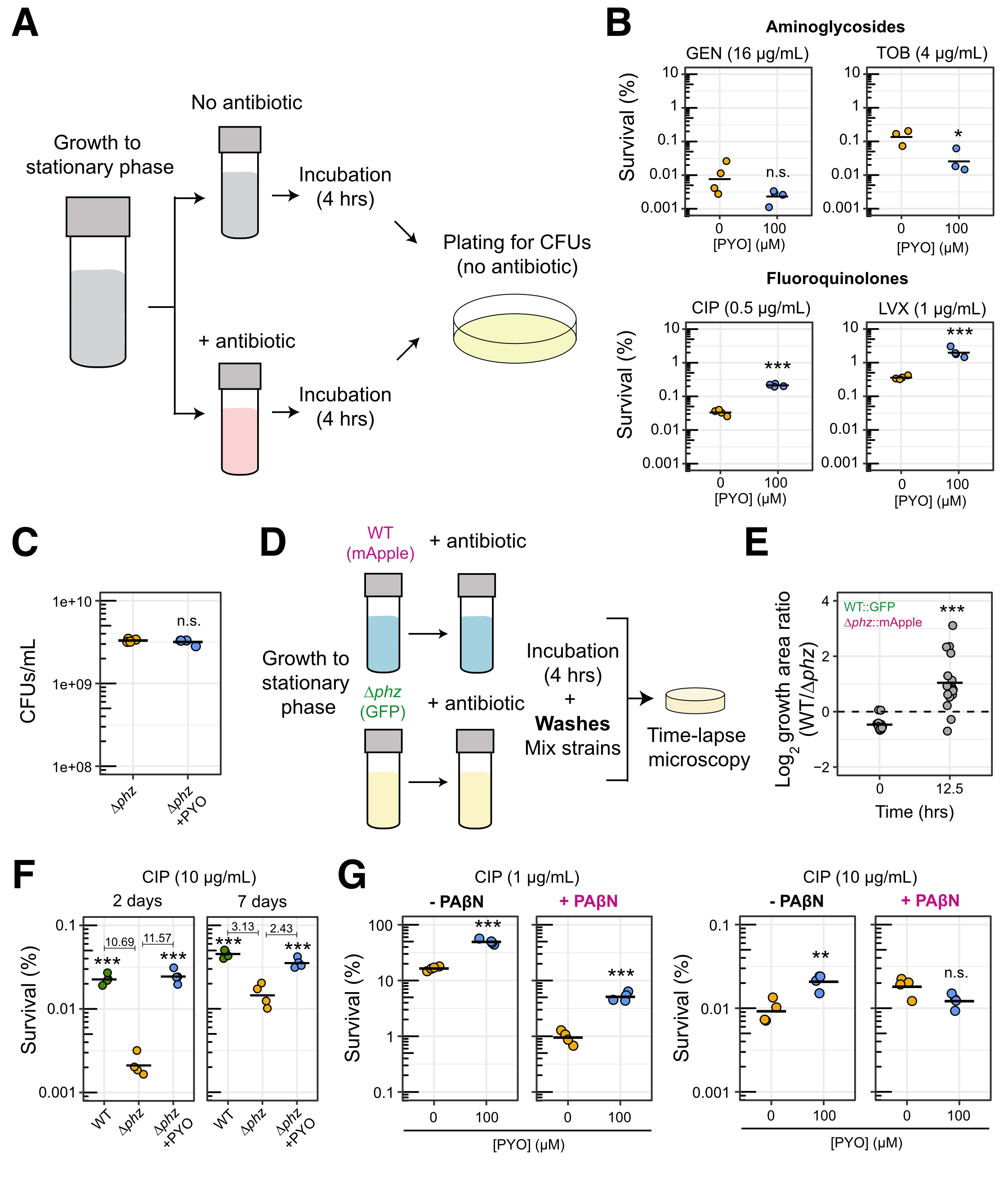

Supplement: S4 Fig — (A) Experimental design for survival assay to measure tolerance to clinical antibiotics. In conditions with exogenous PYO, the PYO was added when cultures were inoculated. PYO itself was not lethal under these experimental conditions (see panel C in this Fig). (B) Tolerance levels of Δphz cells harvested in log phase, following growth in GMM in the presence or absence of PYO (100 μM), to different aminoglycosides and fluoroquinolones (GEN, TOB, CIP, and LVX). Data points represent replicates (n = 4 for all except tobramycin, for which n = 3). Stationary-phase tolerance experiments in GMM are not shown for the aminoglycosides (GEN and TOB), as treatment with TOB in stationary phase under our conditions at this clinically relevant concentration [32] did not result in cell death, regardless of the presence of PYO. However, for experiments performed with stationary-phase cells in SCFM, killing did happen (see Fig 2D). (C) Representative data showing CFUs counted for Δphz grown for 20 hours (in GMM, see Methods) in the presence and absence of PYO in our tolerance assays, showing that PYO itself was not toxic under the studied conditions (n = 4). These are the CFUs for the negative control (no antibiotic) for the experiment performed with CIP in Fig 2C. (D) Experimental design for time-lapse microscopy experiments, in which cells were grown on agarose pads after exposure to CIP (10 μg/mL) in GMM. The strain/fluorescent protein examples shown (i.e., WT::mApple, Δphz::GFP) are the ones used in the images of Fig 2F and S1 Movie. (E) Quantification of microscopy data as done in Fig 2G, but for the experiment with swapped fluorescent proteins. (F) Experiment quantifying how PYO affects lag for CFUs appearing after treatment with CIP (10 μg/mL, see Methods) in GMM. Treating P. aeruginosa cells with 10 μg/mL resulted in high killing levels (see panel G), and we observed an increased lag in the absence of PYO (this supports microscopy data presented in Figs 2F and 2G and S4E Fig) [file pbio.3001093.s004.tif]

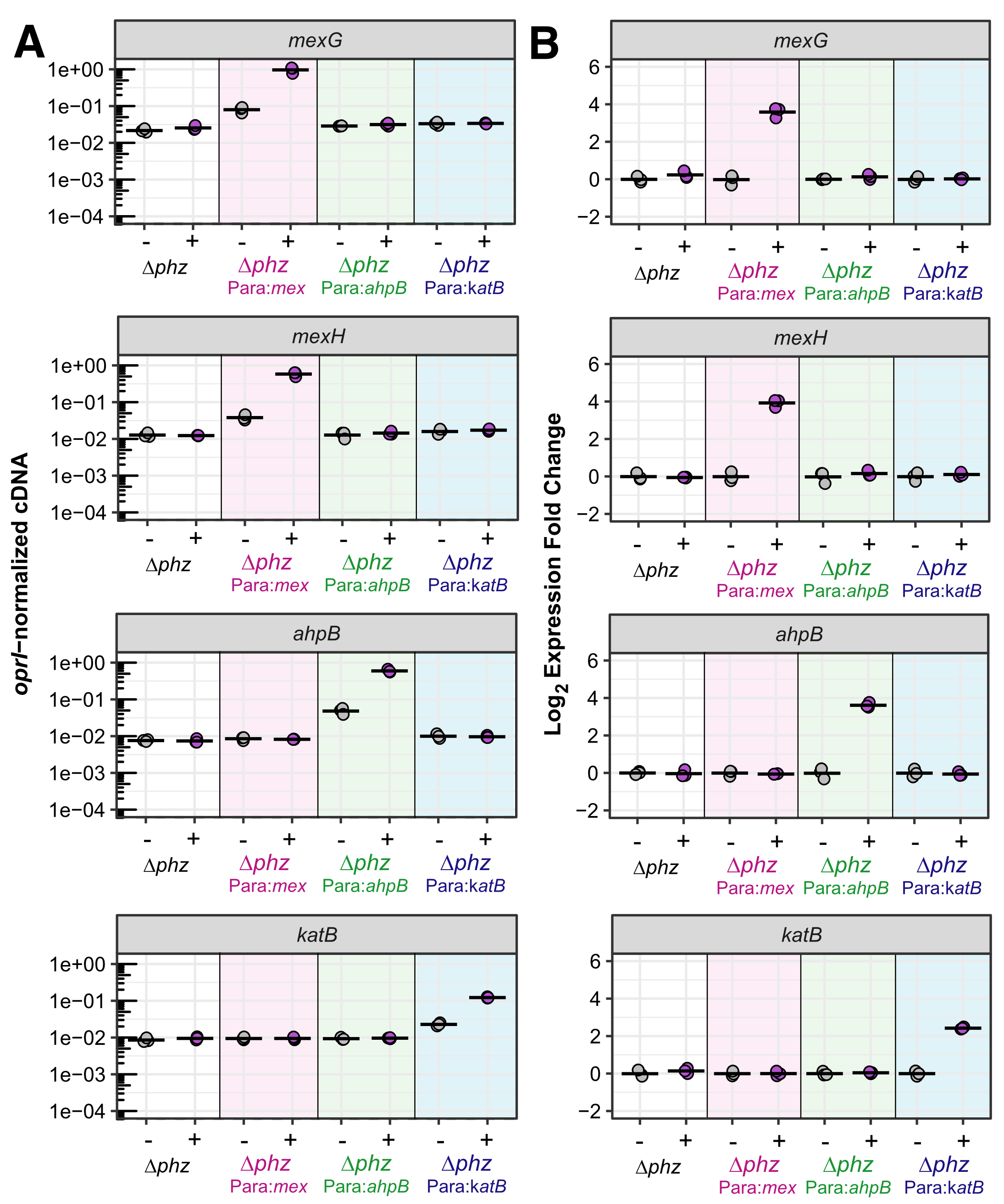

Supplement: S5 Fig — (A) Normalized cDNA levels measured by qRT-PCR. cDNA levels were normalized by the housekeeping gene oprI. (B) Fold change in expression upon arabinose induction. This dataset can be compared to the PYO-mediated induction of the same genes as shown in Fig 2B and S1–S3 Figs. The 4 strains shown are (1) the parent Δphz (white background); (2) Δphz Para:mexGHI-opmD (magenta background); (3) Δphz Para:ahpB (green background); and (4) Δphz Para:katB (blue background). +/− represent addition or not of 20 mM arabinose to the cultures for the artificial induction of expression. For additional experimental details and strain information, see Methods and S5 Table. Black horizontal lines mark the mean value for independent biological cultures (n = 3). The data underlying this figure can be found in Table J in S1 Data. PYO, pyocyanin; qRT-PCR, quantitative reverse transcriptase PCR. (TIF) [file pbio.3001093.s005.tif]

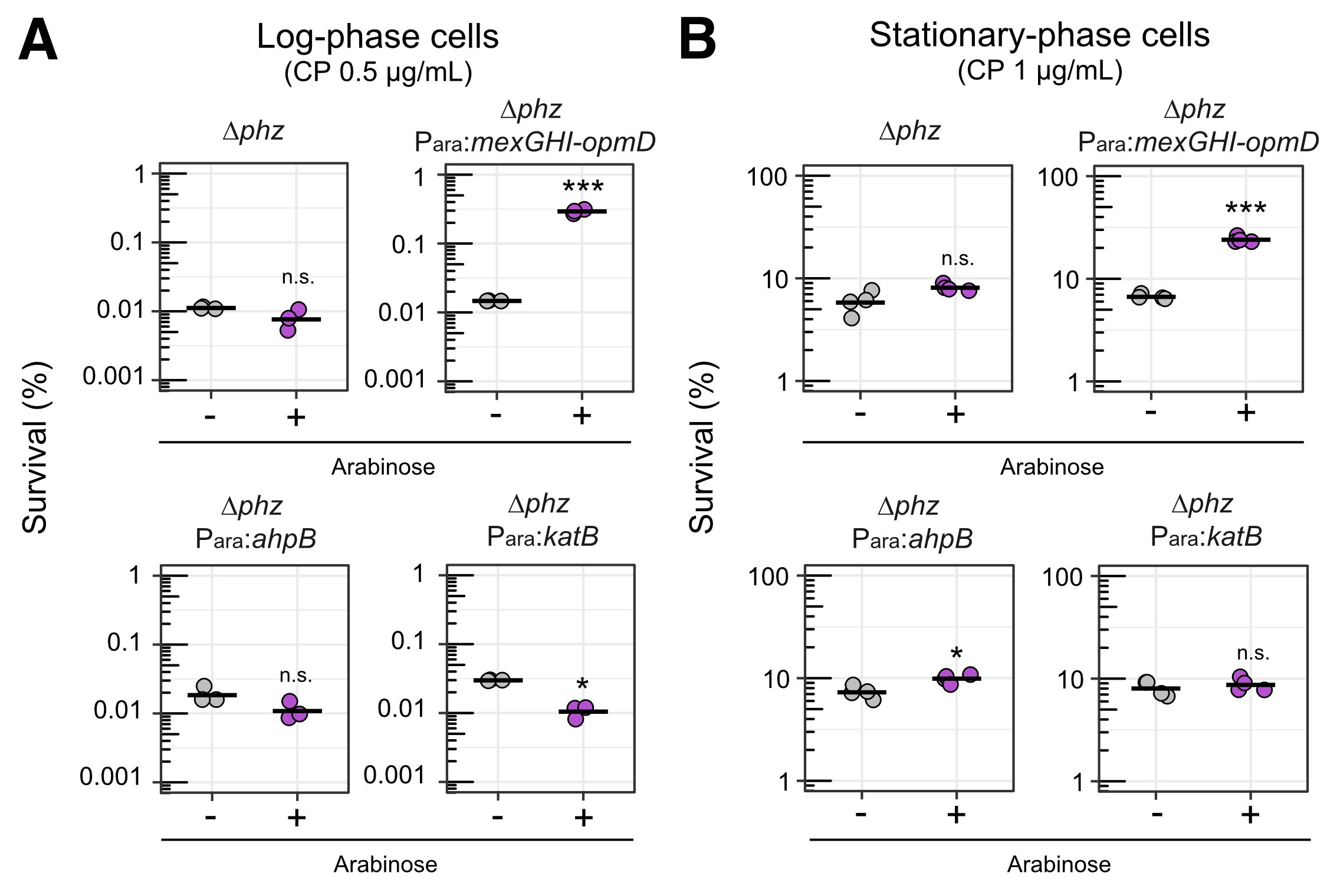

Supplement: S6 Fig — Survival relative to the no-antibiotic control is shown for the parent Δphz strain and the 3 arabinose-inducible strains (in which the PYO-inducible genes mexGHI-opmD, ahpB, or katB are under control of an arabinose-inducible promoter) grown in the presence or absence of 20 mM arabinose, without exposure to PYO. The tolerance experiments were performed for cultures in both log phase (A, n = 3) and stationary phase (B, n = 4) in GMM. In B, the experiment for mexGHI-opmD is the same as in Fig 2I but is also shown here for ease of comparison. Statistics: Welch unpaired t tests (* p < 0.05, ** p < 0.01, *** p < 0.001, n.s. p > 0.05). Black horizontal lines mark the mean value for independent cultures. The data underlying this figure can be found in Table K in S1 Data. CIP, ciprofloxacin; GMM, glucose minimal medium; PYO, pyocyanin. (TIF) [file pbio.3001093.s006.tif]

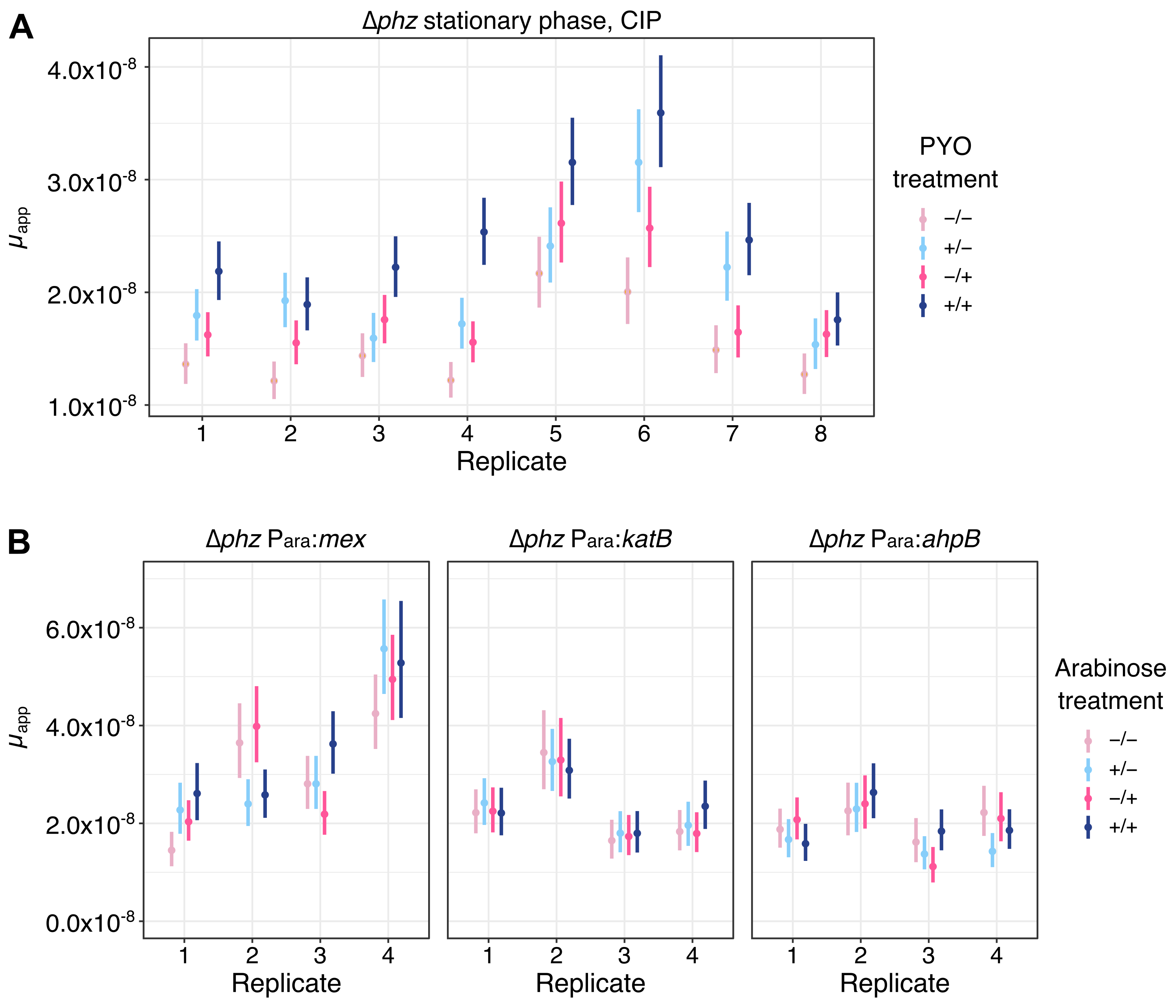

Supplement: S7 Fig — (A) Apparent mutation rates of stationary-phase Δphz grown in liquid GMM and plated onto MH agar containing CIP (0.5 μg/mL), with or without pre- and/or co-exposure to 100 μM PYO relative to the antibiotic selection step (n = 8). (B-C) Apparent mutation rates of log-phase cells grown in GMM and plated onto MH agar containing CIP (0.5 μg/mL), with or without pre- and/or co-exposure to 20 mM arabinose relative to the antibiotic selection step. Data are shown for Δphz Para:mexGHI-opmD alone (B, left, n = 4) and in comparison to Δphz (B, right) or Δphz Para:katB and Δphz Para:ahpB (C, n = 4). In all panels, each data point represents 44 parallel cultures from a single biological replicate, and the vertical lines represent 84% confidence intervals, for which lack of overlap corresponds to statistical significance at the p < 0.05 level [99]. The PYO treatments correspond to the following: −/− denotes no PYO pretreatment (in the liquid culture stage) or co-treatment (in the antibiotic agar plates), +/− denotes PYO pretreatment but no co-treatment, −/+ denotes PYO co-treatment without pretreatment, and +/+ denotes both PYO pretreatment and co-treatment. The data underlying this figure can be found in Table L in S1 Data. CIP, ciprofloxacin; GMM, glucose minimal medium; MH, Mueller–Hinton; PYO, pyocyanin. (TIF) [file pbio.3001093.s007.tif]

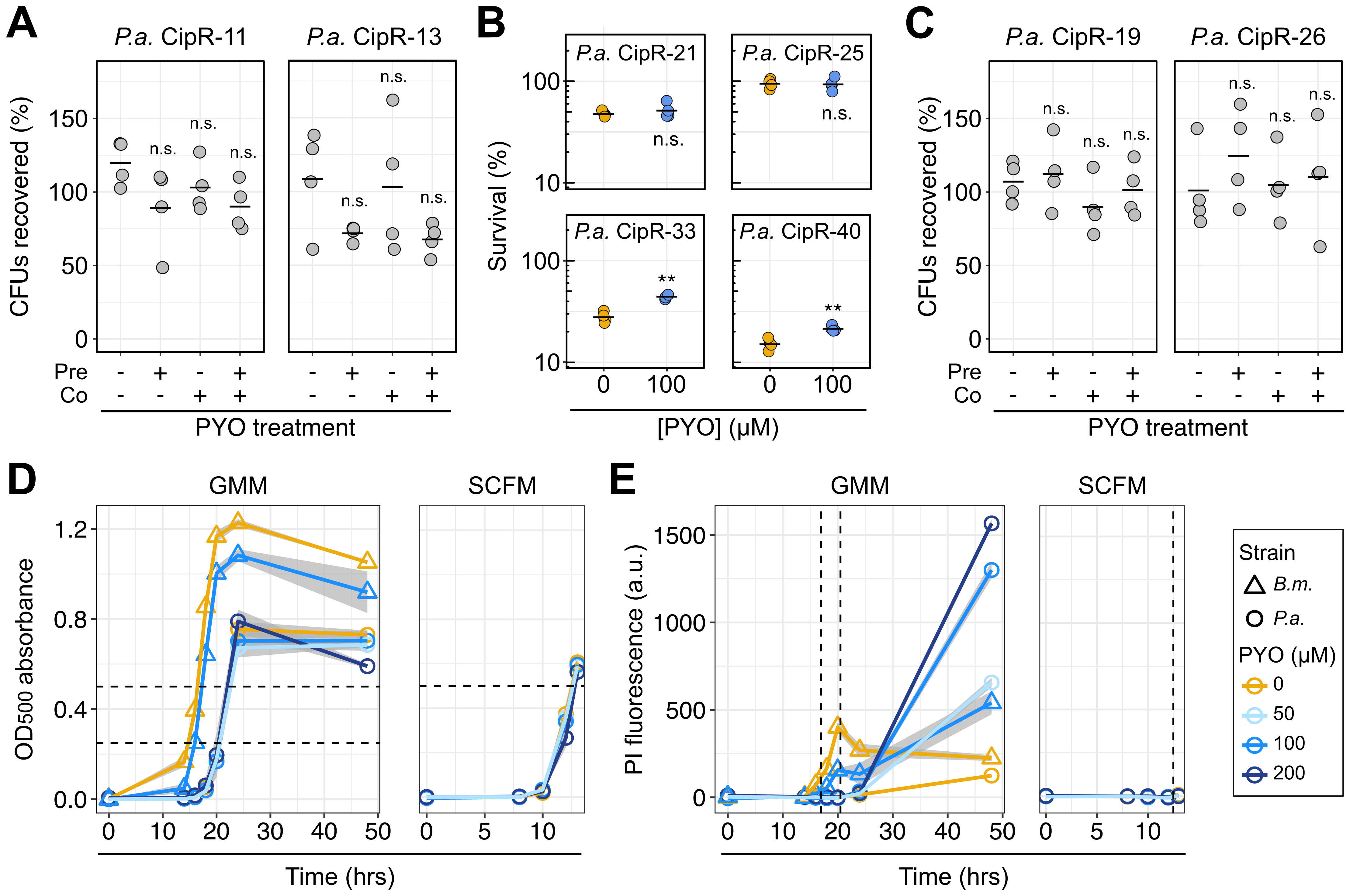

Supplement: S8 Fig — (A) The percentage of CFUs recovered on CIP (0.5 μg/mL) either with or without 100 μM PYO in the agar, for log-phase cultures of representative resistant mutants of P.a. that were not enriched by exposure to PYO in the fluctuation tests. The mutants were pre-grown with or without 100 μM PYO in GMM before plating. On the x-axis, “pre” denotes the presence of PYO in the liquid cultures and “co” denotes the presence of PYO in the agar plates. Percentage recovery was calculated relative to total CFUs counted on nonselective plates (n = 4). (B) Tolerance to CIP (1 μg/mL) of partially resistant mutants grown in GMM to stationary phase with or without 100 μM PYO (n = 4). Experiments were performed as shown in S5A Fig. (C) The percentage of CFUs recovered on CIP (0.5 μg/mL) for log-phase cultures of representative resistant mutants that were enriched by exposure to PYO in the fluctuation tests (n = 4). Experiments were performed in the same way as in panel A. (D, E) Growth curves performed for P. aeruginosa Δphz (P.a.) and B. multivorans 1 (B.m.) in GMM (with the addition of AA for B. multivorans; see Methods) or SCFM, with different concentrations of PYO in the presence of 5 μM PI, which is a fluorescent marker for cell death. OD500 (cell density) is plotted in G, while PI fluorescence is plotted in H. Gray shaded regions represent the standard deviation of 4 biological replicates. In G, the dashed horizontal lines mark the cell density at which P. aeruginosa (lower line in left panel) or B. multivorans (upper line in left panel) would have been plated in our fluctuation tests. Note that these OD500 values differ from those reported in the Methods section for fluctuation tests due to the use of a microtiter plate reader in this experiment, whereas a different spectrophotometer was used in the fluctuation tests. In H, the vertical dashed lines mark the time at which the cultures would have been plated in the fluctuation tests (in the left panel, left line = B.m. sampling ti [file pbio.3001093.s008.tif]

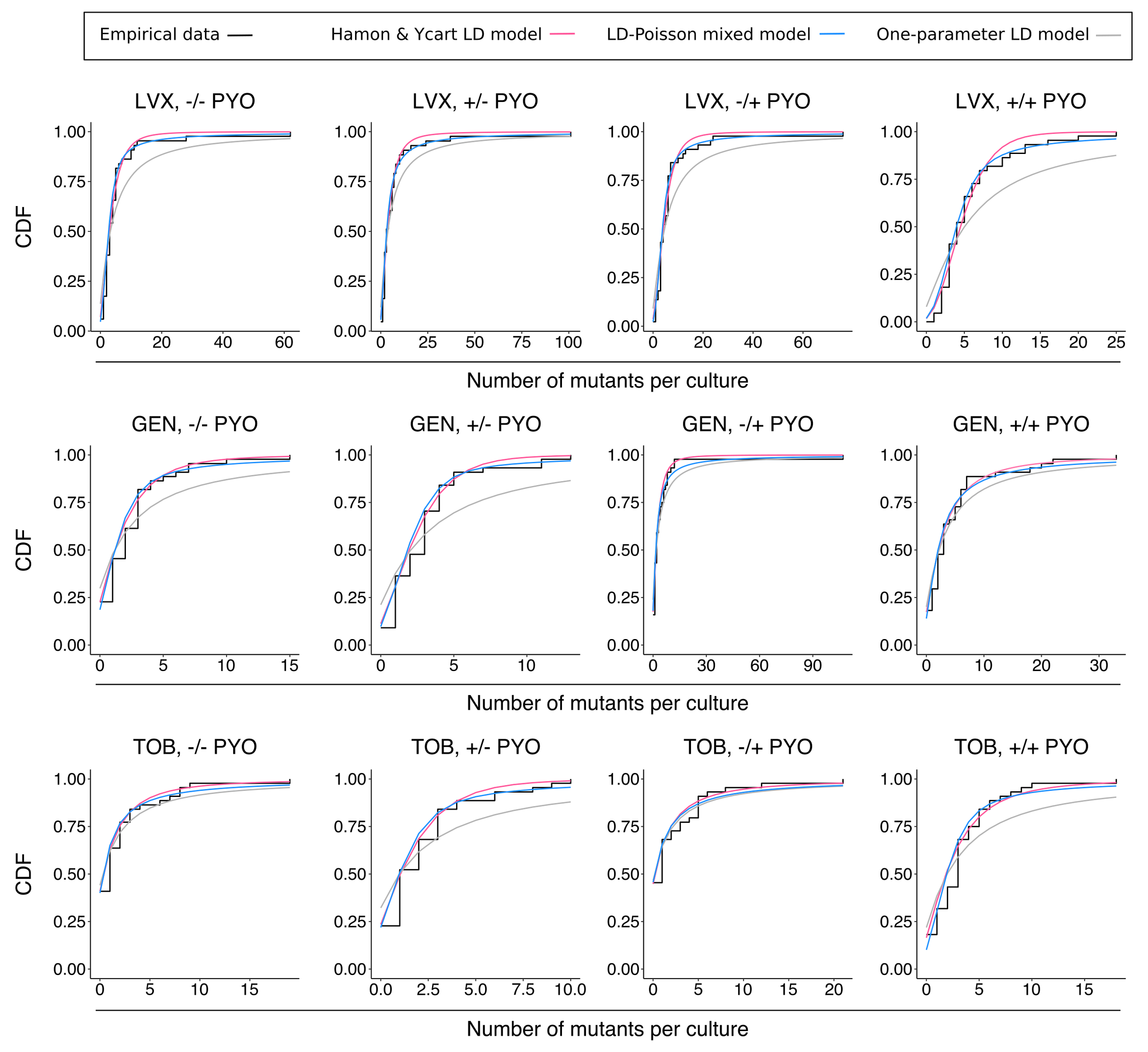

Supplement: S9 Fig — Data are plotted for different combinations of PYO in liquid (pretreatment) and PYO in agar (co-exposure to antibiotic selection). The empirical CDFs of the data (black) are plotted against (1) a variation of the LD model fit with 2 parameters, m (the expected number of mutations per culture) and w (the relative fitness of mutant cells vs. WT), as implemented by Hamon and Ycart [44] (pink); (2) a mixed LD and Poisson distribution fit with 2 parameters, m and d (the number of generations that occur post-plating), allowing for the possibility of post-plating mutations, as implemented by Lang and colleagues [45] (blue); (3) the basic LD distribution model fit only with m, as implemented by Lang and colleagues [45] (gray). In each condition, the plotted data represent the biological replicate with the lowest chi-squared goodness of fit p-value (i.e., least good fit) for the Hamon and Ycart model. The data underlying this figure can be found in Table N in S1 Data. CDF, cumulative distribution function; GEN, gentamicin; LD, Luria–Delbrück; LVX, levofloxacin; PYO, pyocyanin; TOB, tobramycin; WT, wild-type. (TIF) [file pbio.3001093.s009.tif]
